# Variability of intervertebral joint stiffness between specimens and spine levels

**DOI:** 10.3389/fbioe.2024.1372088

**Published:** 2024-02-29

**Authors:** Samuele L. Gould, Giorgio Davico, Christian Liebsch, Hans-Joachim Wilke, Luca Cristofolini, Marco Viceconti

**Affiliations:** ^1^ Biomechanics Group, Department of Industrial Engineering, Alma Mater Studiorum—University of Bologna, Bologna, Italy; ^2^ Medical Technology Lab, IRCCS Istituto Ortopedico Rizzoli, Bologna, Italy; ^3^ Institute of Orthopaedic Research and Biomechanics, Centre for Trauma Research Ulm, Ulm University Medical Centre, Ulm, Germany

**Keywords:** spine, intervertebral joint, multibody modelling, musculoskeletal modelling, personalization, lumbar, tissue characterization, inter-specimen variability

## Abstract

**Introduction:** Musculoskeletal multibody models of the spine can be used to investigate the biomechanical behaviour of the spine. In this context, a correct characterisation of the passive mechanical properties of the intervertebral joint is crucial. The intervertebral joint stiffness, in particular, is typically derived from the literature, and the differences between individuals and spine levels are often disregarded.

**Methods:** This study tested if an optimisation method of personalising the intervertebral joint stiffnesses was able to capture expected stiffness variation between specimens and between spine levels and if the variation between spine levels could be accurately captured using a generic scaling ratio. Multibody models of six T12 to sacrum spine specimens were created from computed tomography data. For each specimen, two models were created: one with uniform stiffnesses across spine levels, and one accounting for level dependency. Three loading conditions were simulated. The initial stiffness values were optimised to minimize the kinematic error.

**Results:** There was a range of optimised stiffnesses across the specimens and the models with level dependent stiffnesses were less accurate than the models without. Using an optimised stiffness substantially reduced prediction errors.

**Discussion:** The optimisation captured the expected variation between specimens, and the prediction errors demonstrated the importance of accounting for level dependency. The inaccuracy of the predicted kinematics for the level-dependent models indicated that a generic scaling ratio is not a suitable method to account for the level dependency. The variation in the optimised stiffnesses for the different loading conditions indicates personalised stiffnesses should also be considered load-specific.

## 1 Introduction

Musculoskeletal multi-body models (MSK) of the human spine allow for investigations, such as soft tissue characterisation, which would present ethical and practical challenges *in vivo* (Wang et al., 2021). Additionally, MSK models allow for investigating situations which could present a risk to human subjects (e.g., large loads on the cervical spine) (Silvestros et al., 2019). The study by Silvestros *et al.* showed a proof-of-concept application to investigate injury mechanisms of the cervical spine using an MSK model (Silvestros et al., 2019). Beaucage-Gauvreau *et al.* developed a model to allow for the non-invasive investigation of lower back loads during lifting activities (Beaucage-Gauvreau et al., 2019). Other studies have investigated ways of improving surgical treatments by simulating different instrumentation strategies for the same patient (La Barbera et al., 2021). Further, MSK models allow for sensitivity analyses, such as the effect of intervertebral joint (IVJ) location and soft tissue properties on the compressive loads that the vertebrae experience (Senteler et al., 2016; Byrne et al., 2020). They, therefore, have the potential to reduce the risk of spinal injuries and improve spinal surgery outcomes (Beaucage-Gauvreau et al., 2019; Gould et al., 2021; Dall’Ara et al., 2022), and so reduce the cost to individuals and society.

The passive soft tissues between adjacent vertebrae (i.e., the intervertebral joint and ligaments), commonly referred to as the IVJ, have different mechanical properties which contribute to the functional behaviour of the spine and are crucial to maintaining spinal stability (Widmer et al., 2020). MSK models of the spine often simplify the IVJ to three rotational degrees of freedom (DoF) with a fixed centre of rotation (Petit et al., 2004; Han et al., 2012), although more recent studies have included translational DoF (Meng et al., 2015; Ignasiak et al., 2016; Arshad et al., 2017). The mechanical properties of the IVJs are not included in all models and if present are simplified to a lumped parameter spring-damper model, commonly referred to as a bushing force (Silvestros et al., 2019; Wang et al., 2019; 2021; Alizadeh et al., 2020; Gould et al., 2021). Parameters for the bushing force are taken from the literature (Han et al., 2012; Christophy et al., 2013; Ignasiak et al., 2016; Senteler et al., 2016; Arshad et al., 2017; Zhang et al., 2020; Remus et al., 2021; Lerchl et al., 2022) and applied in each DoF (Silvestros et al., 2019; Wang et al., 2019; 2021; Alizadeh et al., 2020; Gould et al., 2021). Despite these simplifications, representing the IVJ with a bushing force is complex due to the interaction between joint pose and stiffness which influences the predicted joint loads and muscle forces (Byrne et al., 2020). Furthermore, although rarely accounted for, the IVJ properties vary strongly between specimens and spine levels in all DoF (Panjabi et al., 1976; Andersson and Schultz, 1979; Miller et al., 1986; McGlashen et al., 1987; Izambert et al., 2003; Petit et al., 2004; Renner et al., 2007; Garges et al., 2008; Doulgeris et al., 2014; Schmidt et al., 2015; Newell et al., 2019; Palanca et al., 2020) and influence predicted muscle forces, intervertebral disc loads, and range of motion (Arshad et al., 2017; Wang et al., 2019; Byrne et al., 2020). Moreover, several studies have highlighted the importance of determining subject- or specimen-specific and condition-specific stiffnesses which can result in more accurate kinematic predictions (Ghezelbash et al., 2016; Alizadeh et al., 2020; Wang et al., 2020; 2021). Therefore, accurate subject-specific characterisation of the IVJ is necessary to prove the reliability of models.

Personalisation of the IVJ stiffness has been done using hybrid models (Remus et al., 2021) and by using finite element models which are integrated into MSK models (Byrne et al., 2020). Another method to estimate subject-specific properties has been to scale the stiffness based on anthropometric data (Ghezelbash et al., 2016). The meta-analysis of studies of the rotational behaviour of human cadaveric spine segments by Zhang *et al.* has established a regression model of the moment-rotation behaviour of the IVJ (Zhang et al., 2020) which could be used to assign stiffnesses to the IVJ in MSK models. However, few studies have directly optimised the stiffness within MSK models. Wang *et al.* developed a generalised stiffness model from literature data of the IVJ which was incorporated into an MSK model (Wang et al., 2020), using this model they went a step further, optimising a subject-specific stiffness based on *in vivo* motion (Wang et al., 2021). One study has used a genetic algorithm with MSK simulations to optimise the IVJ stiffness of the model for *ex vivo* porcine cervical spine specimens (Silvestros et al., 2019). These studies considered the level dependency of the IVJ stiffness at the individual IVJ levels (Silvestros et al., 2019; Wang et al., 2021). To simulate spinal surgeries Petit *et al.* developed a method using functional bending tests to optimise patient-specific stiffnesses, however, this approach simplified the level dependency by dividing the thoracolumbar spine into three regions (Petit et al., 2004).

Stiffnesses have been personalised in flexion/extension and lateral bending under flexion/extension motion and lateral bending motion using motion capture data from *in vivo* experiments, personalised stiffnesses in axial rotation were not reported (Wang et al., 2021). Given the direction-dependent nature of the IVJ stiffness (Miller et al., 1986; McGlashen et al., 1987; Renner et al., 2007) further work is needed to predict stiffnesses under axial rotation. Furthermore, optimising the stiffnesses in the rotational DoFs required an estimation of the joint kinematics using an optimisation algorithm as the motion capture data came from *in vivo* experiments (Wang et al., 2021). Wang *et al.* suggested that with more accurate motion tracking data, stiffnesses could be optimised without calculating the joint kinematics through optimisation, this is possible with data from *ex vivo* experiments (Silvestros et al., 2019) but has not been done for rotational DoFs.

While previous studies have shown optimisation of the subject or specimen-specific stiffnesses improves the accuracy of the spinal MSK models (Silvestros et al., 2019; Wang et al., 2021), the aims of the previous studies were not to investigate the inter-specimen variability or the variation between spine levels of optimised stiffnesses. A study focusing on the inter-specimen and spine level variability which optimises stiffnesses within MSK models is lacking and would evidence the need for specimen-specific optimisation which accounts for IVJ level dependency and loading conditions. Moreover, a study using highly accurate motion tracking in combination with MSK models to simultaneously optimise the stiffnesses of the lumbar spine in all rotational DoF for multiple loading conditions would strengthen and build upon the existing studies.

Therefore, the current study aimed to investigate the variability of optimised stiffnesses across different specimens and different spinal levels in all rotational DoF through numerical simulations using accurate motion capture data collected *in vitro* from human specimens to allow for highly accurate tracking. This also included investigating these aspects under different loading.

## 2 Materials and methods

**FIGURE 1:**
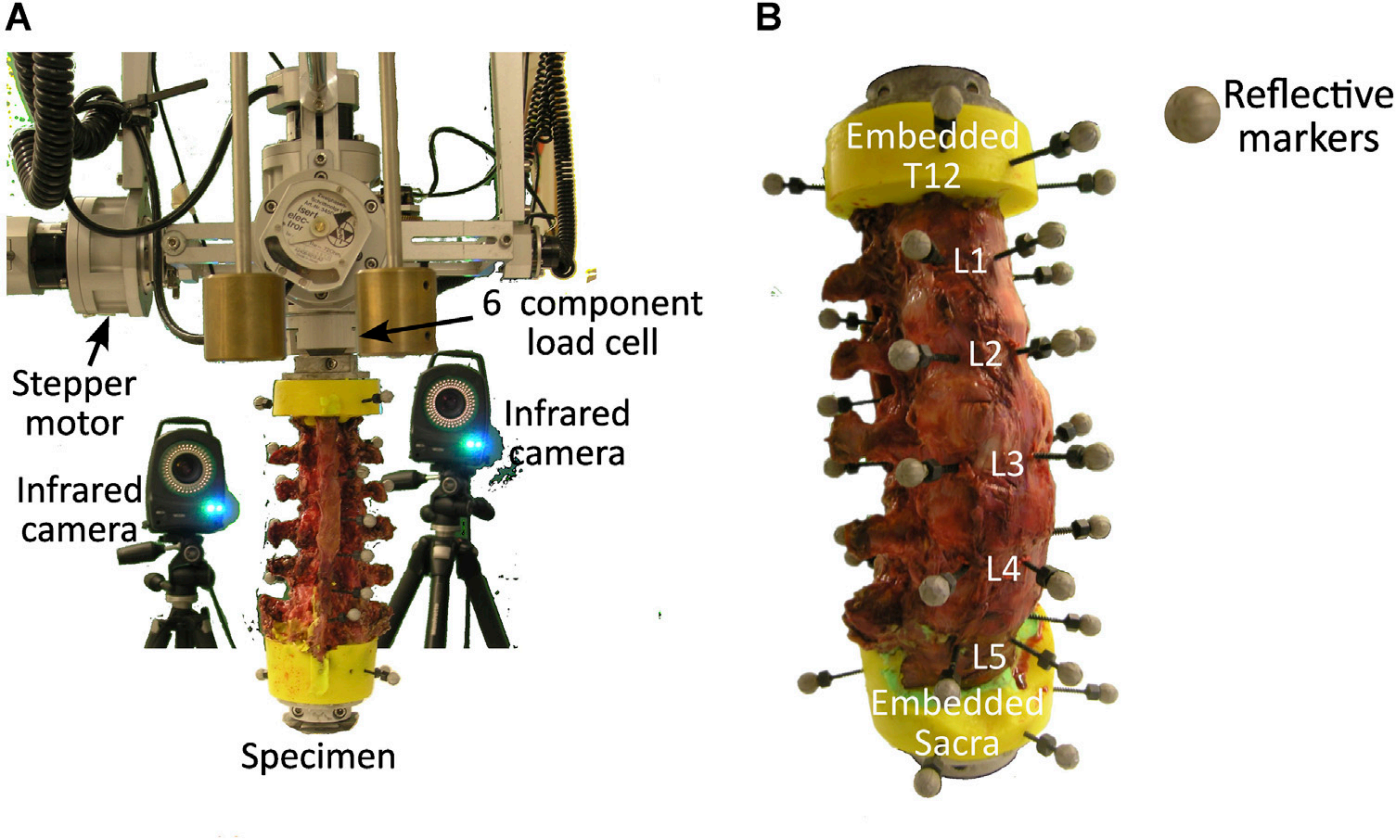
**(A)** Experimental setup showing the specimen held in place by a lower fixture and the load cell above which attaches to the stepper motors, and two of the infrared cameras. **(B)** Anterior view of a specimen with three reflective markers attached to the anterior surface of each vertebra.

This study reanalysed a subset of the imaging, load, and motion capture data from the results of the *in vitro* biomechanical study by Volkheimer *et al.* (Volkheimer et al., 2018). More specifically, the experimental study (Volkheimer et al., 2018) provided data for six T12-sacrum spine segments from human cadavers (Table 1). The specimens were acquired from the Science Care (United States) donation program. In brief, the specimens were cleaned of soft tissue leaving the vertebrae, intervertebral discs, all ligaments, and facet joints intact. The sacrum was completely constrained and pure moments were applied to T12 using a spine tester (Wilke et al., 1994). Three loading conditions were individually applied, flexion/extension, lateral bending, and axial rotation. Each loading condition was applied as a pure moment by means of a gimbal and stepper motor (Wilke et al., 1994). At all joints there were 6 DoF, therefore although a pure moment was applied, the nature of spinal biomechanics meant that the joints experienced coupled moments. 3.5 loading cycles were applied at 1.0°/s in flexion/extension and lateral bending and 0.5°/s in axial rotation with a peak moment of 7.5 Nm. The loading rates were selected to reduce the influence of creep and inertia on the results and the load magnitude was based on recommendations within literature (Wilke et al., 1998). The load cycle for evaluation was selected as the one with the smallest range of the coupled moments. The first cycle was excluded *a priori* as the initial recorded load was not always 0N. The moment in the loaded DoF and the resulting coupled moments in the non-loaded DoF were measured using a six-component load cell mounted above the specimens (FT 1500/40, Schunk GmbH, Lauffen/Neckar, Germany) (Figure 1A). Three reflective markers were attached to the anterior surface of each vertebra (Figure 1B) (Volkheimer et al., 2018). The vertebral motion was measured with a motion tracking system (Vicon MX13+, Vicon Motion Systems Ltd., Oxford, UK) including six infrared cameras. From this, the rotation in each DoF at each joint was calculated, the measured rotations were in agreement with other *in vitro* studies (Panjabi et al., 1994). These rotations were then provided as inputs for the current computational study. Additionally, the specimens were imaged twice:

• CT scans with a pixel size of 0.39 × 0.39 mm and slice increment of 0.5 mm (thickness 1.0 mm) were obtained with a Brilliance 64, Philips CT device using a voltage of 120 kVp and a tube current of 356 mA,
• A planar X-ray of each specimen mounted in the experimental setup was provided from a lateral view (source—AJEX 140H, Ajex Meditech Co., Ltd., Gyeonggi-do, Republic of Korea; digital cassette—FCR standard cassette, 14″ × 17″, Fujifilm Holdings Corporation, Tokyo, Japan).
• The experimental study was not originally intended to be used as the input for the current computational study, therefore the CT scans were not performed with the markers attached to the vertebrae, whereas they were visible in the planar X-Ray.

**TABLE 1:** Specimen details.

Specimen	Age	Sex	Height (m)	Mass (kg)	Cause of death
1	57	Female	1.57	59	Metastatic lung cancer
2	54	Female	1.57	36	Malignant colon cancer
3	56	Female	1.72	48	Melanoma
4	59	Female	1.65	113	Lung carcinoma/COPD
5	44	Female	1.63	49	Cardiac arrest
6	49	Male	1.72	59	Lung cancer

### 2.1 Workflow overview

**FIGURE 2:**
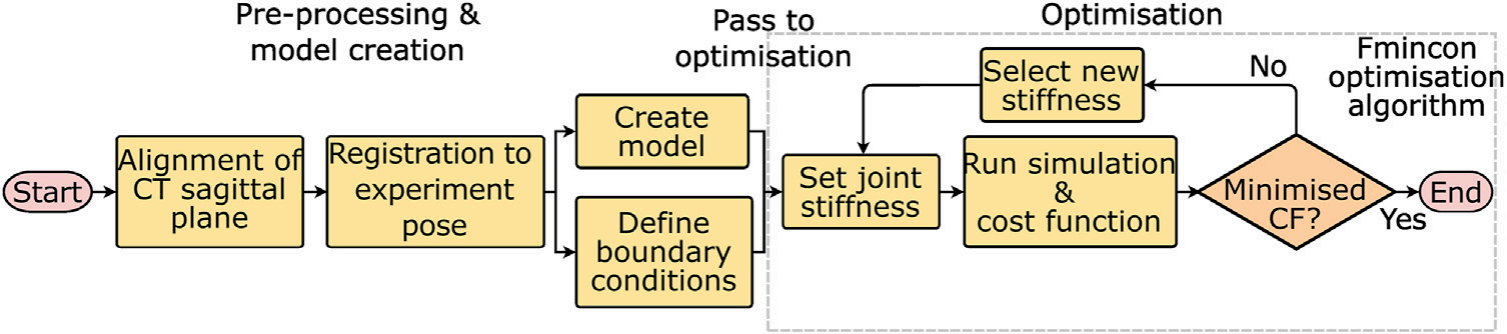
Workflow for optimising the stiffnesses starting from the experimental data. CF = cost function, and fmincon is the optimisation algorithm used to minimise the cost function.

The workflow for optimising the stiffnesses will be discussed in two stages, first pre-processing and model creation, and second optimisation (Figure 2). The pre-processing required two steps, the first was an alignment of the CT data to the sagittal plane of the spine. This was followed by a registration procedure. Then the models and boundary conditions could be defined and passed to the optimisation process which optimised the stiffnesses.

### 2.2 Alignment of CT data to the sagittal plane of the spine

**FIGURE 3:**
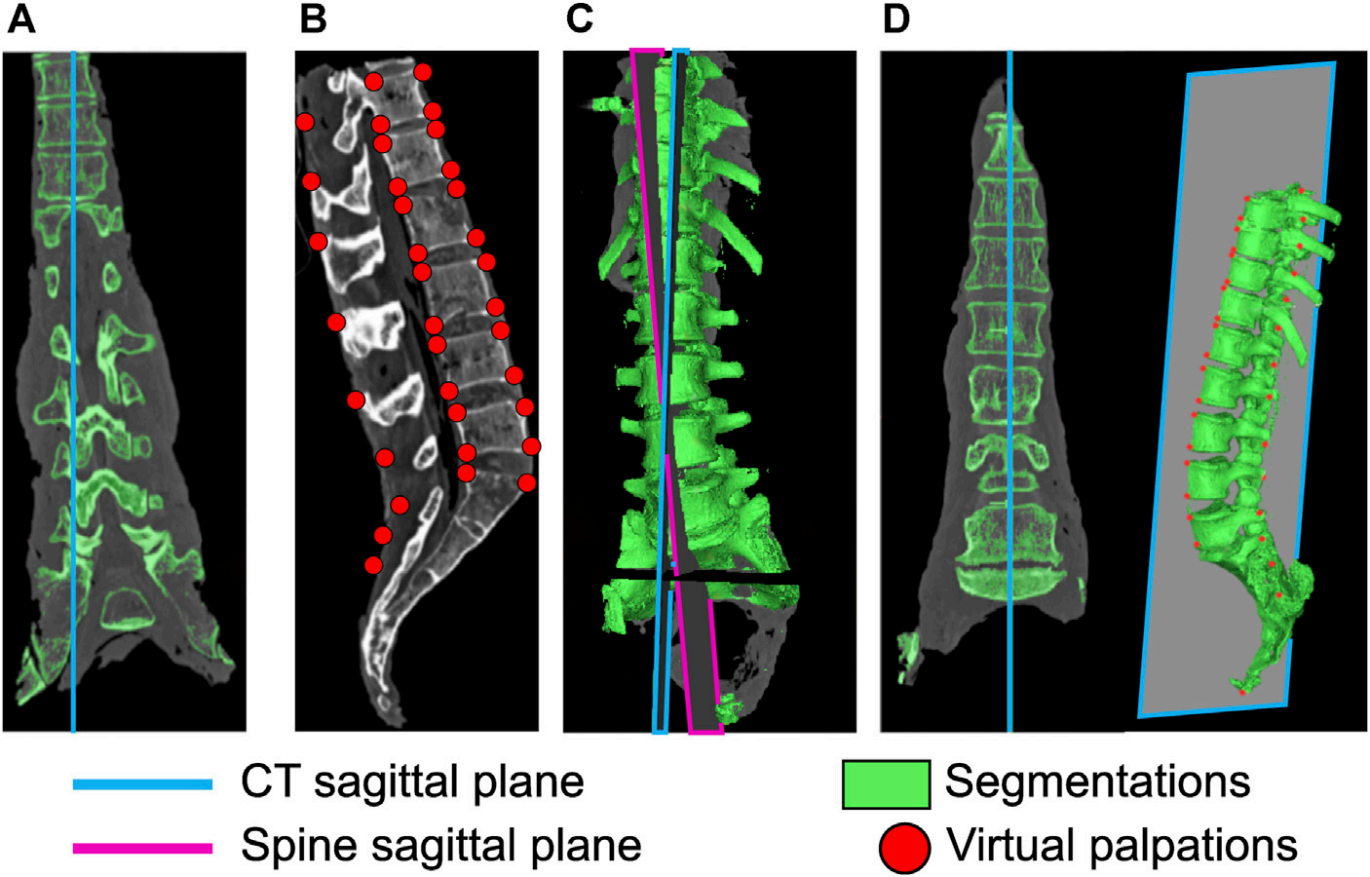
Realignment of the CT sagittal plane. **(A)** original sagittal plane of the CT data, **(B)** virtual palpation of landmarks defining the spine sagittal plane, **(C)** definition of the spine sagittal plane and the sagittal plane of the CT data, **(D)** CT data after realigning the CT sagittal plane to the spine sagittal plane.

The specimens were imaged in different poses (both globally, and also possibly with different inter-vertebral angles) in the CT scanner and with the X-ray during the experiment. To ensure the model was in the same pose as the physical specimen, the CT data needed to be registered to the X-ray taken during the experiment. To do this the X-ray image was assumed to be aligned with the sagittal plane of the spine which was defined by a set of landmarks on the 3D geometry. However, as the sagittal, frontal, and transverse planes of the CT data were not aligned with the corresponding planes of the spine (Figure 3A), an alignment procedure was necessary. A four-step process (see Supplementary Data SA for details) was followed:

1. In Mimics (Mimics Innovation Suite v24, Materialise, Leuven, Belgium)—virtual palpation (applying markers to anatomical landmarks on a medical image or computer model) of the CT data was performed to identify landmarks on the sagittal plane of the spine (Figure 3B).
2. A custom script in MatLab (MatLab R2021b, The Mathworks, Natick, MA, United States)—a plane was fitted to these landmarks to define the sagittal plane of the spine.
3. In Mimics—the sagittal plane of the spine is defined (Figure 3C).
4. In Mimics—CT data is resampled along the sagittal plane of the spine (Figure 3D).

### 2.3 Registration of CT data to X-ray

After the re-alignment of the CT data, the vertebral bodies were reconstructed via manual segmentation, and two virtual palpations were performed. One set of virtual palpations identified the landmarks necessary to define the IVJ pose following the ISB recommendations (Wu et al., 2002) (Supplementary Data SA). The second set enabled the registration of the CT data to the X-ray (Supplementary Data SA), hereafter referred to as CT registration landmarks.

To register the CT data to the X-ray acquired during the experiment, the X-ray was virtually palpated with markers placed on the anterior-most and posterior-most points of the inferior and superior endplates. Where this was unclear, multiple markers were placed and the average was taken.

The CT registration landmarks were projected onto the sagittal plane of the spine. The CT registration landmarks were moved into the same reference system as the X-ray markers. The X-ray markers were scaled using an estimation from the Euclidean distance of the markers on the endplates. The estimation was necessary as the X-ray was originally intended for grading the state of disc degeneration, therefore quantitative information regarding the field of view, the pixel size and detector element dimension was not available.

The rotational components of the transformation matrices to perform the registration on each vertebra were calculated using the average angle of the superior and inferior endplates. The translational components of the transformation matrices for the registration were calculated based on the centre of the markers on each vertebra. A transformation matrix was defined for each vertebra, which was then applied to the segmentation of each vertebra and the associated joint markers (Supplementary Data SA). The joint markers were then used to define the joint pose following the ISB recommendations (Wu et al., 2002).

### 2.4 Model creation and simulation

**FIGURE 4:**
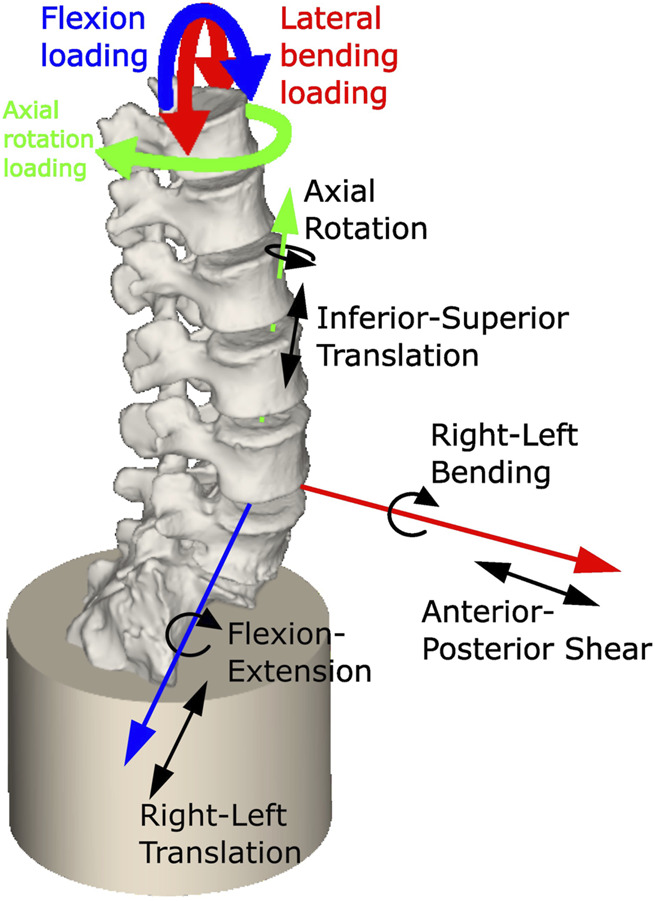
Model showing constrained sacrum, the loading conditions applied, and a single joint with the allowed degrees of freedom (others are not shown for clarity but have the same DoF).

An OpenSim model of each specimen was created using a custom MatLab script and the OpenSim API (code provided at OpenSim project Specimen specific spine models). In the models, the sacrum was fully constrained. A 6 DoF joint was created for each IVJ in the pose previously defined (Figure 4). A bushing force (spring-damper element) was defined as coincident with the joint in each DoF, with generic stiffness values taken from the literature for the L3L4 IVJ level (Table 2)(Senteler et al., 2016). These stiffnesses were different in each DoF but uniform across the joint levels, these models will be referred to as Models_Lit_Unif_. A uniform stiffness was applied to reduce the cardinality for the optimisation routine (reducing the computational expense) as optimising the stiffnesses at each level in all rotational DoF would require a cardinality of 18. This was necessary as a preliminary optimisation for a single model with a cardinality of 3 lasted >8 h. Damping parameters of 1000 N/(m/s) were used for the translational dampers and 2.3 Nm/(rad/s) for the rotational dampers were taken from the literature (Jager, 1996).

**TABLE 2:** The initial rotational and translational stiffness for the uniform stiffness models (Models_Lit_Unif_) and the level-dependent models (Models_Lit_Lev_Dep_). The translational stiffnesses were the same for the uniform and the level-dependent models as no scaling factor was applied to the translational stiffnesses.

Spinal level	Rotational stiffnesses, Nm/rad		
	Right-left bending	Axial rotation	Flexion/extension
Uniform	68.8	291	51.0
T12L1	36.4	136.8	16.3
L1L2	43.6	128.1	27.5
L2L3	19.9	291.1	36.7
L3L4	12.4	291.1	49.5
L4L5	13.8	291.1	50.0
L5S1	68.8	183.4	51.0

	Translational Stiffnesses, Nm		
	Anterior-Posterior Shear	Inferior-Superior Translation	Right-Left Translation
Uniform	149,000	1,890,000	135,000

The approach of uniform stiffness across the joints represents an overall spine stiffness. To also investigate the representation of the variation of stiffnesses across the joints, a set of models which incorporated level-dependent stiffnesses were also created (Table 2). These models are referred to as Models_Lit_Lev_Dep_ (Table 2). To introduce the level-dependency, a scaling factor was applied to the rotational stiffnesses. The scaling factor was calculated as the ratio between stiffnesses based on the data used for fitting the regression models in the meta-analysis by Zhang *et al* (Zhang et al., 2020).

Quasi-static loading conditions were used to replicate the low loading rate of the experiment. Preliminary simulations using fully dynamic loading conditions found a single iteration could take over an hour, and an optimisation typically required over 200 iterations. The loading cycles were analysed to determine the loads to apply to the model. The first cycle was excluded as the initial recorded load in the first cycle was not always 0N. The other cycles were examined to identify the one with the smallest range of the coupled moments. The maximum torque in that cycle and the corresponding coupled moments were applied for the loading cycle duration to T12 to simulate flexion, left axial rotation, and left lateral bending (Figure 4). The kinematics corresponding to the selected cycle were extracted from the joint kinematic data.

### 2.5 Optimisation of the intervertebral joint stiffness

A custom MatLab script and the OpenSim API used these boundary conditions and the models with stiffnesses from literature (Models_Lit_Unif_ and Models_Lit_Lvl_Dep_) to run forward dynamic simulations within an interior-point optimisation algorithm (*fmincon*). In each loop, the optimisation algorithm optimised the stiffness parameters in all rotational DoF to minimise the kinematic error. For the optimisations using Models_Lit_Unif_, uniform stiffness across the joints was maintained, the optimised models are referred to as Models_Optim_Uni_. For the optimisations using Models_Lit_Lvl_Dep_, the ratio between the levels was maintained, the optimised models are referred to as Models_Optim_Lvl_Dep_. The optimiser sought to minimise the sum of the squared motion tracking error (Eq. 1).

$$cf=\sum_{i=1}^{n}{\left(p_{i}-m_{i}\right)}^{2}$$ (1)

Where *i* was the DoFs included in the cost function, which went up to *n* rotational DoFs (two or three depending on the loading direction), *p*_*i*_ was the predicted motion, and *m*_*i*_ was the measured motion.

Optimisations were performed for each loading condition (lateral bending, axial rotation, and flexion). In all scenarios, all rotational DoF stiffnesses were optimised. The cost function was sensitive to the tracking error of different DoF depending on the loading direction. Therefore, for each loading condition, the cost function minimised the motion errors for the DoF to which it was sensitive. Under a left lateral bending load, this corresponded to flexion and lateral bending; under an axial rotation load this corresponded to flexion, lateral bending, and axial rotation; and under a flexion load, this corresponded to flexion and lateral bending. This corresponded to the DoF in which the loading was applied, as in this DoF there was the largest motion. Then the other rotational DoF were included if the measured motion in these DoF was of the same order of magnitude as the largest motion.

### 2.6 Validation and analysis of results

#### 2.6.1 Cross-validation

A cross-validation using the leave-one-out technique was performed for both sets of optimised models (Models_Optim_Unif_ and Models_Optim_Lvl_Dep_) in each loading condition. As six specimens were modelled, this resulted in a total of six cross-validations. To perform each cross-validation, the cross-validation model was assigned the median stiffness of the other five optimised models and subsequently employed to simulate the relevant loading condition. The stiffness was not further optimised. The cross-validation was performed for the uniform stiffness models (Models_CV_Unif_) and the level-dependent models (Models_CV_Lvl_Dep_). This process was repeated for each of the six models under the relevant loading condition. A total of 36 simulations (two stiffness representations for six specimens for three loading conditions).

#### 2.6.2 Variation between specimens

To assess the inter-specimen variability, the median and range of the optimised stiffnesses for each specimen in Models_Optim_Unif_ and Models_Optim_Lvl_Dep_ were evaluated. The difference in the prediction accuracy of the specimens and the corresponding cross-validation models were evaluated using the normalised root mean square error (RMSE) of all joint levels in all DoF. Statistically significant differences were identified by running a Kruskal-Wallis test, sampling, from each specimen, the error of all the joints in the DoF in which the load was applied. The Kruskal-Wallis test was chosen to evaluate the differences between specimens because the RMSE of the specimens were assumed to be independent of each other and the data was non-parametrically distributed. Each test used a sample size of six (corresponding to the six specimens). This test was performed for Models_Optim_Unif_ and Models_Optim_Lvl_Dep_, resulting in a total of 36 comparisons.

#### 2.6.3 Variation between spine levels

To investigate the importance of accounting for the variation of stiffness between spine levels, Kruskal-Wallis tests were performed to evaluate any statistically significant differences in the errors between joint levels. The Kruskal-Wallis test was chosen to evaluate the differences between joint levels because the joint levels were assumed to be independent of each other as the RMSE was being considered and the data was non-parametrically distributed. Each test used a sample size of five (corresponding to the five joint levels). The errors in the DoF in which the load was applied were used in the test (e.g., under a flexion load, errors in the flexion DoF were analysed) as it was in that direction that the largest motion occurred. These tests were performed for Models_Optim_Unif_ and Models_Optim_Lvl_Dep_. Additionally, the RMSE of the joint levels were compared.

#### 2.6.4 Comparison of generic and optimised stiffnesses

To test for statistically significant improvements following the optimisation, Wilcoxon signed-rank tests were performed on the kinematic errors for each individual model pre- and post-optimisation (Models_Lit_Unif_ vs. Models_Optim_Unif_) and then for post-optimisation and the validation models (Models_Optim_Unif_ vs. Models_CV_Unif_). Wilcoxon signed-rank tests were considered to be the most suitable statistical test because the pre- and post-optimisation models of the same specimen are not independent of each other, and the data was non-parametrically distributed. Each test had a sample size of 15 (the errors in three DoF for each of the five joint levels), the test was repeated six times (once for each specimen). A Bonferroni correction was applied to account for the multiple tests (Bland and Altman, 1995). This was repeated for each loading condition with Models_Lit_Lvl_Dep_, Models_Optim_Lvl_Dep_, and Models_CV_Lvl_Dep_ for a total of 72 comparisons.

#### 2.6.5 Effectiveness of a ratio level dependency

Finally, to analyse the effectiveness of using a ratio to introduce level dependency, the RMSE of each specimen in the separate DoF and the overall RMSEs (i.e., across all joints and all DoF) of each specimen for Models_Optim_Unif_ and Models_Optim_Lvl_Dep_ were compared under each loading condition for a total of 12 comparisons. The Kruskal-Wallis tested for statistically significant differences between the errors of the two optimised model sets (Models_Optim_Uni_ and Models_Optim_Lvl_Dep_). The Kruskal-Wallis test was used because Models_Optim_Unif_ and Models_Optim_Lvl_Dep_ are independent of each other and the data was non-parametrically distributed. The tests for lateral bending and flexion loading were performed with a sample size of six while for axial rotation the sample size was four due to the optimisation failing to converge for two of the specimens. The samples for the tests consisted of the RMSE in the separate DoF and the overall RMSE of all models.

For all statistical tests performed the null hypothesis was rejected for *p* < 0.05.

## 3 Results

First, a summary of the optimised stiffnesses and kinematic errors of the three types of models (literature, optimised, and cross-validation) considering the loading direction is presented. Then the overall inter-specimen variability for both the uniform and level-dependent models is discussed. Next, the variation between spine levels is discussed, first addressing the uniform models and then the level-dependent models. Finally, a direct comparison is made between the results of the optimised uniform models and the level-dependent models.

### 3.1 Optimised stiffnesses and kinematic errors

**FIGURE 5:**
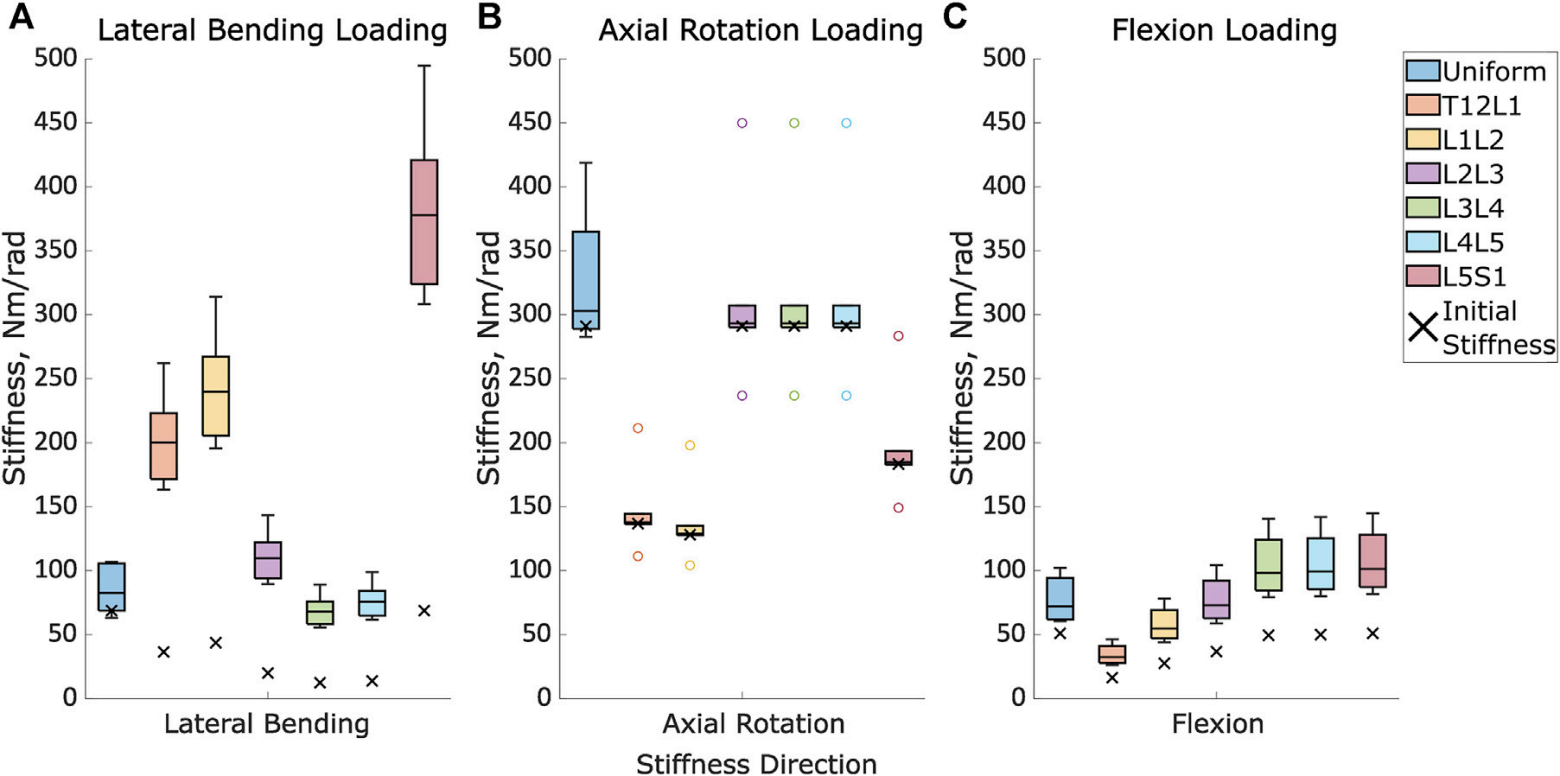
The initial stiffness and the distribution of the optimised stiffnesses for the uniform models and at each intervertebral joint level for the level-dependent models in **(A)**. Lateral bending under lateral loading, **(B)**. Axial rotation under axial rotation loading, and **(C)**. Flexion in flexion loading.

First and foremost, the range of optimised stiffness values was larger for the stiffnesses in the loading direction (lateral bending load, axial rotation load, or flexion load) than in the directions in which the loading was not applied (Table 3; Figure 5, Supplementary Data SB).

**TABLE 3:** The initial stiffness values taken from the literature (Senteler et al., 2016) and the optimised stiffnesses in each DoF for the uniform stiffness models under the different loading conditions. DoF of Stiffness labels in bold indicate the DoF which is in the same direction as the loading. The optimisation algorithm did not converge for specimens 2 and 6 under axial rotation.

Load type	DoF of stiffness	Specimen #						
		Initial	1	2	3	4	5	6
Lateral bending	**Lateral bending, Nm/rad**	68.8	107.0	63.0	77.8	105.5	68.8	87.5
	Axial rotation, Nm/rad	291	291.0	270.4	293.8	289.7	291.1	291.5
	Flexion, Nm/rad	51	49.5	68.0	50.7	50.1	51.0	65.1
Axial Rotation	Lateral bending, Nm/rad	68.8	93.3	-	99.0	101.3	94.9	-
	**Axial rotation, Nm/rad**	291	295.1	-	282.7	418.7	310.9	-
	Flexion, Nm/rad	51	58.5	-	47.2	143.8	18.0	-
Flexion	Lateral bending, Nm/rad	68.8	69.4	79.8	67.3	70.5	59.1	68.6
	Axial rotation, Nm/rad	291	291.1	296.4	291.0	291.3	292.7	291.7
	**Flexion, Nm/rad**	51	61.9	65.2	60.5	79	94.2	102.2

**FIGURE 6:**
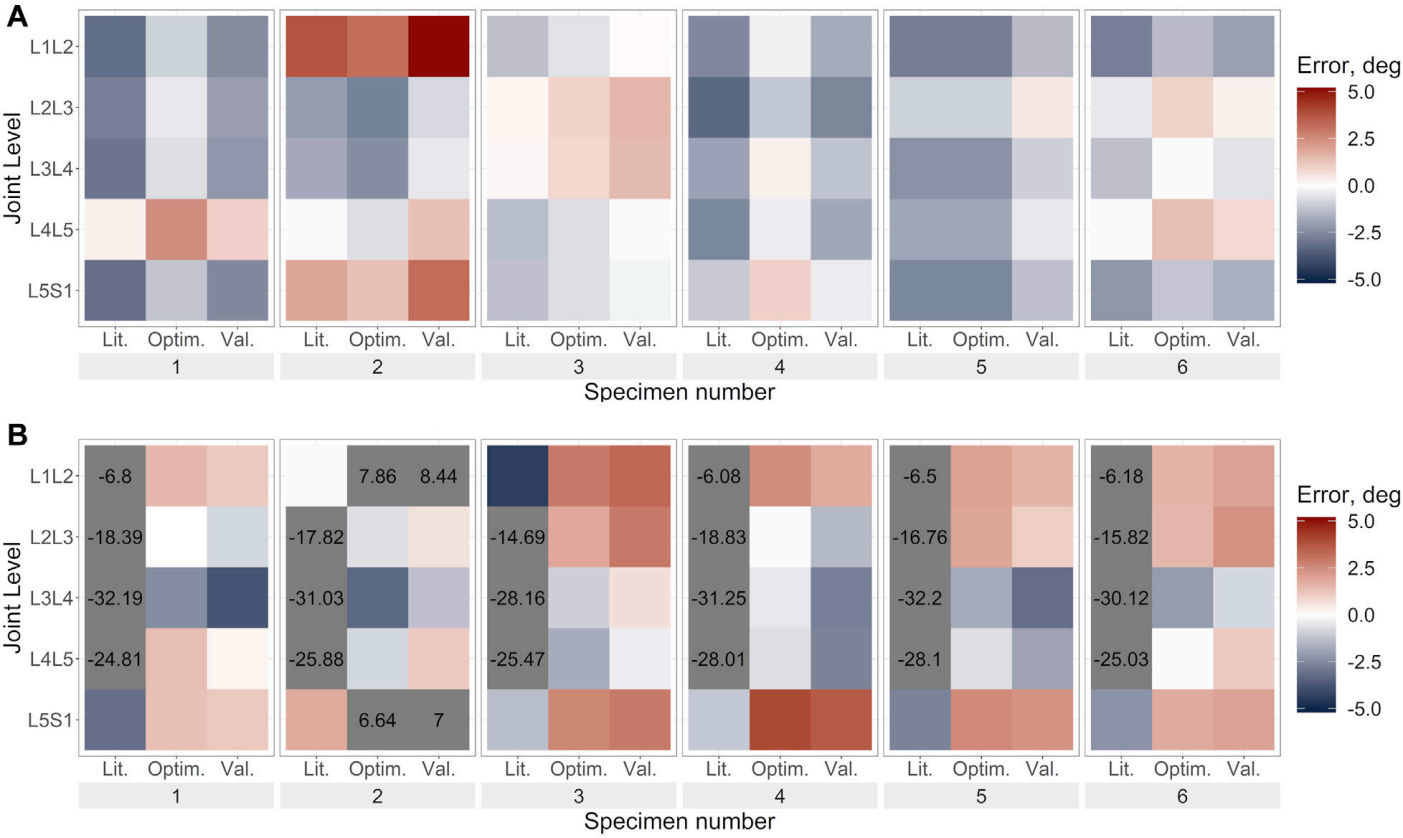
Lateral bending error under lateral bending load for **(A)**. Uniform stiffness models, **(B)**. Level-dependent models for the literature (Lit.), optimised (Optim.) and cross-validation (Val.) stiffnesses. Blue indicates an underprediction of the motion (i.e., too much rotation) while red indicates an overprediction of the motion, and grey indicates levels where the error exceeded the range imposed on the colour scale for clarity.

**FIGURE 7:**
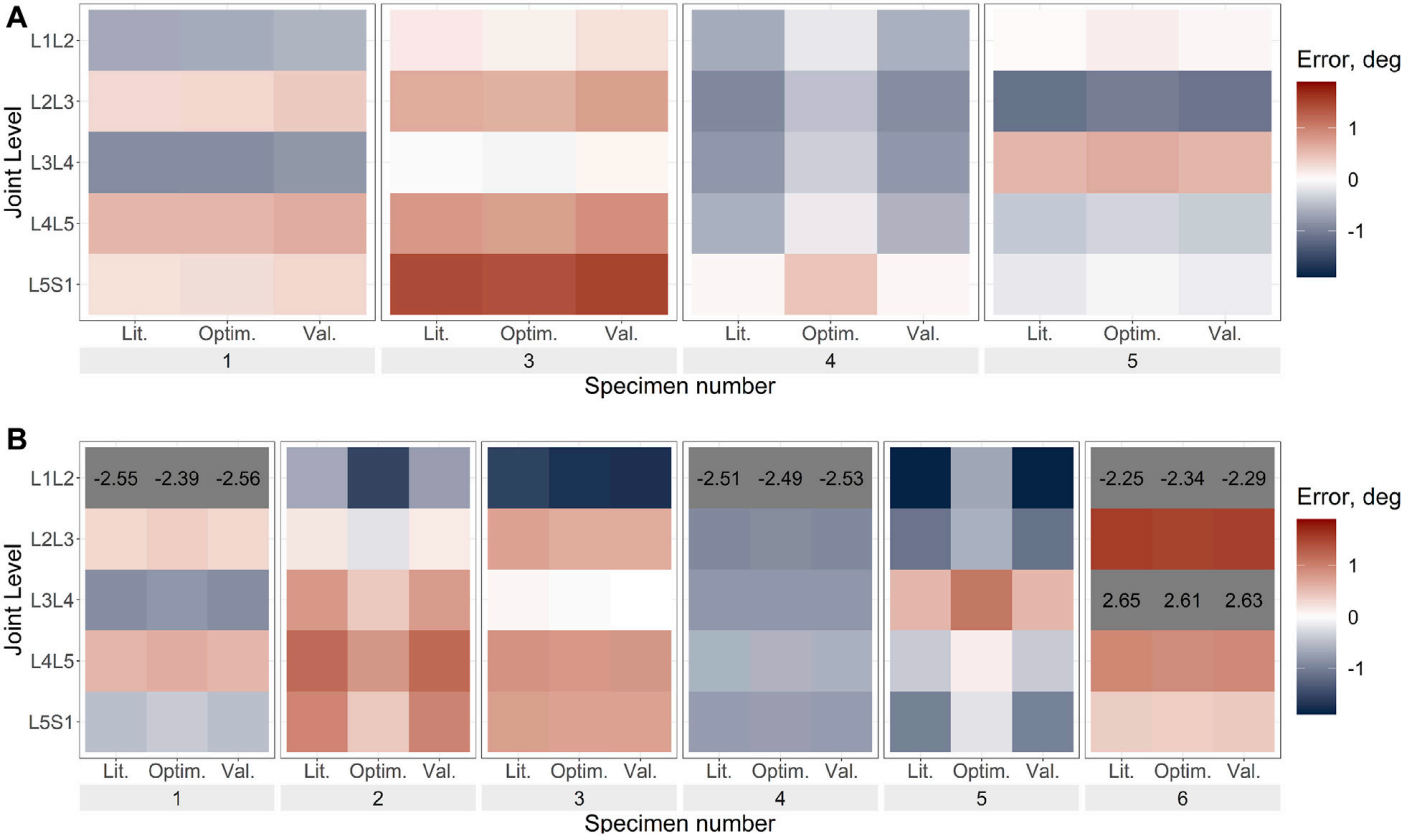
Axial rotation error under axial rotation load for **(A)**. Uniform stiffness models, **(B)**. Level-dependent models for the literature (Lit.), optimised (Optim.) and cross-validation (Val.) stiffnesses. Blue indicates an underprediction of the motion (ie too much rotation) while red indicates an overprediction of the motion, and grey indicates levels where the error exceeded the range imposed on the colour scale for clarity. The optimisation algorithm for the uniform stiffness models was unable to converge for specimens 2 and 6 hence they are not present in **(A)**.

**FIGURE 8:**
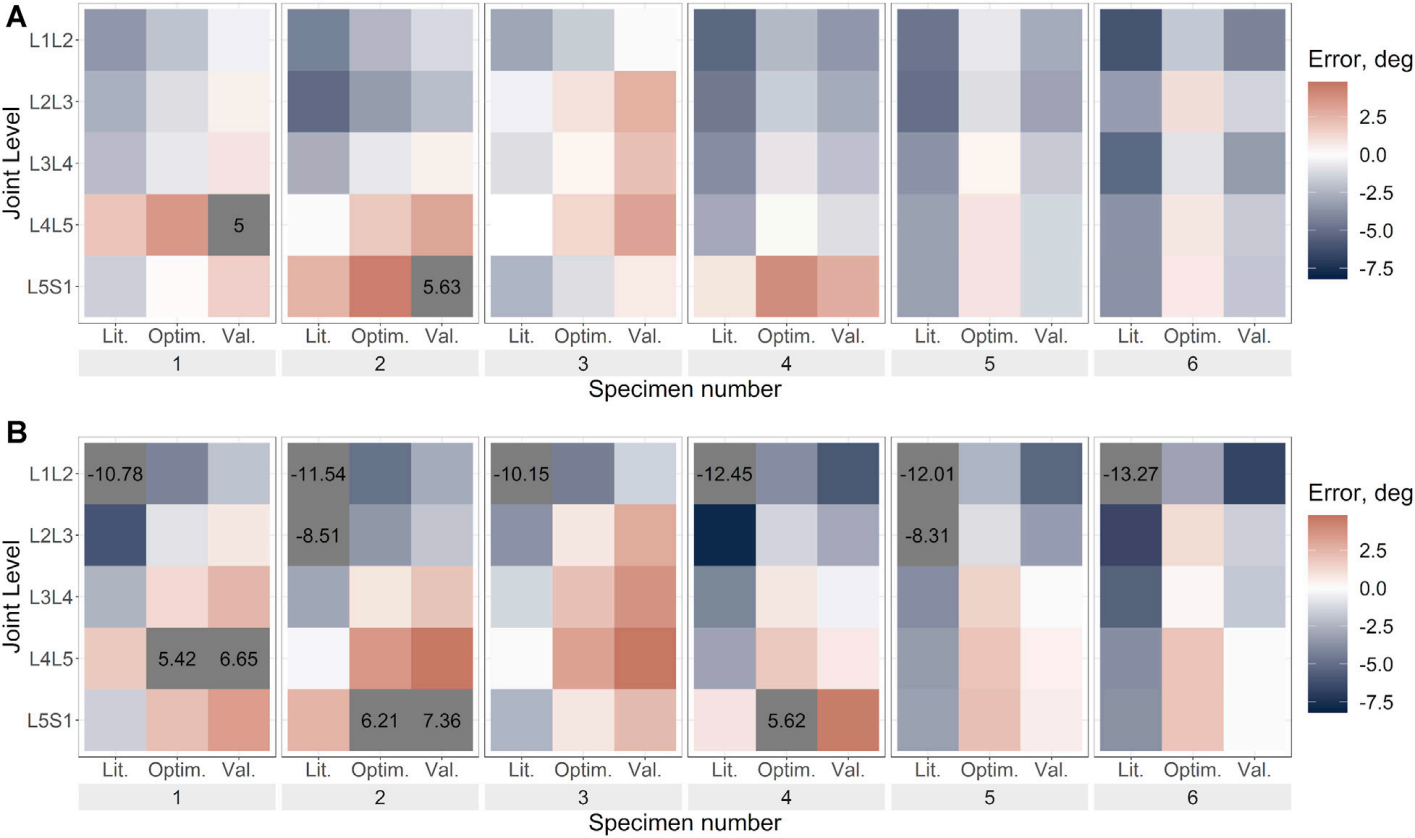
Flexion error under flexion load for **(A)**. Uniform stiffness models, **(B)**. Level-dependent models for the literature (Lit.), optimised (Optim.) and cross-validation (Val.) stiffnesses. Blue indicates an underprediction of the motion (ie too much rotation) while red indicates an overprediction of the motion, and grey indicates levels where the error exceeded the range imposed on the colour scale for clarity.

The prediction errors were lower for the optimised stiffness models than the literature stiffness models and the cross-validation models in all DoF, however, the improvement in the prediction error was largest in the DoF corresponding to the loading direction (Figures 6–8; Supplementary Data SC).

### 3.2 Inter-specimen variability

#### 3.2.1 Uniform stiffness models

The results of the optimised stiffness of the specimens showed a large range relative to the initial stiffness values. Under a lateral bending load the optimised stiffness had a range of 44 Nm/rad (64% of the initial value). Under an axial rotation load, the optimised stiffness had a range of 136 Nm/rad (47% of the initial value). Under a flexion load, the optimised stiffness had a range of 40 Nm/rad (78% of the initial value) (Table 3).

Although the optimisation algorithm was able to converge in most cases when level dependency was not introduced, there were few exceptions. For specimen 5 under lateral bending loads, the stiffness values did not vary. For specimens 2 and 6, under axial rotation, the optimisation algorithm was unable to converge.

The substantial changes in the stiffness pre- and post-optimisation (Figure 5) reduced the percentage mean absolute error (MAE) of the kinematics by 14% under lateral bending loading, 3% under axial rotation loading, and 27% under flexion loading (Table 4). However, these improvements in the prediction error between the types of models (literature vs. optimised and optimised vs. cross-validation) for each specimen were not consistently significant. Under lateral bending loads, the improvement of the prediction error was statistically significant for most specimens when comparing the prediction errors from the optimised stiffness models to that of the literature and cross-validation stiffness models (Table 6). However, no significant differences were found when comparing the prediction error for the optimised and literature stiffness models of Specimen 5, and the cross-validation stiffness model for Specimen 3 (Table 6). Under axial rotation loading and flexion loading the optimised stiffness models did not result in statistically significant differences in the prediction error compared to the prediction error of the literature and cross-validation stiffness models (with one exception, the optimised stiffness model and literature stiffness model of Specimen 2) (Table 6).

**TABLE 4:** The medians and interquartile ranges of the mean absolute errors (MAE) of the specimens and the median MAEs as a percentage of the maximum mean absolute motions in each direction when loaded in the same direction.

Model type	Loading direction	MAE in the loading direction, °					
		Initial stiffness			Optimised stiffness		
		Median	IQR	Percentage error, %	Median	IQR	Percentage error, %
Uniform stiffness models	Lateral bending	2.0	0.9	30	1.1	1.3	16
	Axial rotation	0.6	0.1	27	0.5	0.2	24
	Flexion	3.2	1.6	44	1.2	0.7	17
Level-dependent models	Lateral bending	16.5	1.7	247	1.6	0.6	25
	Axial rotation	1.0	0.3	38	0.9	0.4	33
	Flexion	5.3	1.6	75	2.4	0.9	33

Even with the optimised stiffnesses the differences between the RMSE of the specimens as a percentage error could be as much as 37%. However, these differences were only significant between specimens when using literature stiffnesses under flexion loading (Table 5). There were no significant differences between specimens for any model type under lateral bending or axial rotation loads (Table 5).

**TABLE 5:** *p*-values from Kruskal-Wallis tests performed on each model type. The analysis tested for significant differences in the predicted error between different joint levels (group errors in all 3DoF simultaneously by joint levels) and for significant differences in the predicted error between specimens (group error in all 3DoF simultaneously by specimen) under each loading condition.

Load type	Intervertebral joint representation	Analysis	Model type		
			Literature	Optimization	Cross-validation
Lateral bending	Uniform	Joint	NS	NS	NS
		Specimen	NS	NS	NS
	Level-dependent	Joint	NS	NS	NS
		Specimen	NS	NS	NS
Axial rotation	Uniform	Joint	NS	NS	NS
		Specimen	NS	NS	NS
	Level-dependent	Joint	NS	NS	NS
		Specimen	NS	NS	NS
Flexion	Uniform	Joint	NS	0.003	0.047
		Specimen	NS	NS	0.004
	Level-dependent	Joint	NS	<0.001	0.001
		Specimen	NS	NS	0.047

#### 3.2.2 Level-dependent models

The overall range of the optimised stiffnesses was larger for the level-dependent models than for the uniform models (Figure 5). The improvement in the prediction error when comparing the predictions of the literature and optimised models was only statistically significant for Specimen 4 under lateral bending loading, Specimen 2 under axial rotation loading, and Specimen 5 under flexion loading (Table 6). No statistically significant differences were found for any of the other specimens.

**TABLE 6:** *p*-values from the Wilcoxon tests with Bonferroni adjustment. For each loading condition, the groups were the errors in all directions for pre-optimisation and the errors in all directions for post-optimisation. Tests were repeated using the groups of the errors in all directions for post-optimisation and the errors in all directions for cross-validation. Load types, LB = lateral bending, AR = axial rotation, F = flexion.

Comparison	Model type		Load type	Specimen #					
				1	2	3	4	5	6
Pre-optimization vs. post-optimisation	Uniform		LB	<0.001	<0.001	0.026	<0.001	NS	<0.001
			AR	NS	NA	NS	NS	NS	NA
			F	NS	0.040	NS	NS	NS	NS
	Level-dependent		LB	NS	0.050	NS	0.026	NS	0.050
			AR	NS	<0.001	NS	NS	NS	NS
			F	NS	NS	NS	NS	0.026	NS
Post-optimisation vs. cross-validation	Uniform		LB	<0.001	<0.001	0.050	<0.001	<0.001	0.002
			AR	NS	NA	NS	NS	NS	NA
			F	NS	NS	NS	NS	NS	NS
	Level-dependent		LB	NS	0.001	<0.001	0.001	NS	0.040
			AR	NS	<0.001	NS	NS	NS	NS
			F	NS	NS	NS	NS	NS	NS

The optimisation of the stiffnesses with level dependency resulted in a greater relative improvement in the prediction accuracy than without level dependency (Table 4). However, the prediction errors of the initial stiffness were much larger than those of the stiffnesses without level dependency. Under axial rotation loading the improvement of the predicted errors with optimised stiffnesses was negligible. Notably, the maximum errors tended to be much higher with level-dependent stiffness (Figures 6B, 7B, 8B) than with uniform stiffnesses (Figures 6A, 7A, 8A).

There were no significant differences in the prediction error between specimens when using the literature models, the optimised models, or the cross-validation models under lateral bending loading or axial rotation loading. Significant differences between specimens were found for the cross-validation models but not for the literature models or the optimised models under flexion loading (Table 6).

### 3.3 Variation between spinal levels

#### 3.3.1 Uniform stiffness models

Under lateral bending and flexion loads, the use of a generic stiffness resulted in an overprediction of the motion at all spine levels. Contrarily, with optimised stiffness values, the motion was underpredicted at some levels (e.g., L1L2, specimen 2) while overpredicted at other levels (e.g., L2L3, specimen 2), without any clear relationship to the spinal level (Figures 6A, 8A). No trends were apparent under axial rotation loading (Figure 7A). The predicted RMSE (of the same joint across all specimens) for the uniform models with an optimised stiffness varied by up to 33% between levels. Analysing the specimens individually, the variation between spine levels in the prediction error for the optimised model without any level dependency showed some specimens had similar prediction errors across all spine levels, while for others the magnitude of the error varied between the spine levels (Figures 6A, 7A, 8A). However, no significant differences in prediction error were found between joint levels under lateral bending loads or axial rotation loads for the literature stiffness models, the optimised stiffness models or the cross-validation stiffness models. Significant differences were only found between joint levels under flexion loads for the optimised stiffness models (Table 5).

#### 3.3.2 Level-dependent models

The range of optimised stiffnesses at the individual IVJ levels was smaller for the level-dependent models than the uniform models, except for T12L1, L1L2, and L5S1 under lateral bending loads (Figure 5). With the optimised stiffness the MAE was higher for level-dependent models than for the uniform models (Table 4). However, for certain specimens at certain joint levels, the prediction error was smaller compared to models without any level dependency, for example, L3L4 for Specimen 6 in the flexion direction under a flexion load (Figures 6B, 7B, 8B). The differences in the errors between levels were found to be statistically significant for the optimised stiffnesses and the cross-validation stiffness models but not the literature stiffness models under a flexion load (Table 5). The differences were not statistically significant under lateral bending loads or axial rotation loads.

### 3.4 Comparison of uniform and level-dependent approaches

The introduction of level-dependent stiffnesses with a ratio resulted in a higher optimised stiffness across most levels compared to the optimised stiffness with a uniform stiffness across all levels (Figure 5). The IQR was larger for Models_Optim_Unif_ than the IQR of Models_Optim_Lvl_Dep_ at the central levels (L2L, L3L4, and L4L5) in lateral bending, at all levels in axial rotation and levels L1L2 and L2L3 in flexion (Figure 5).

**FIGURE 9:**
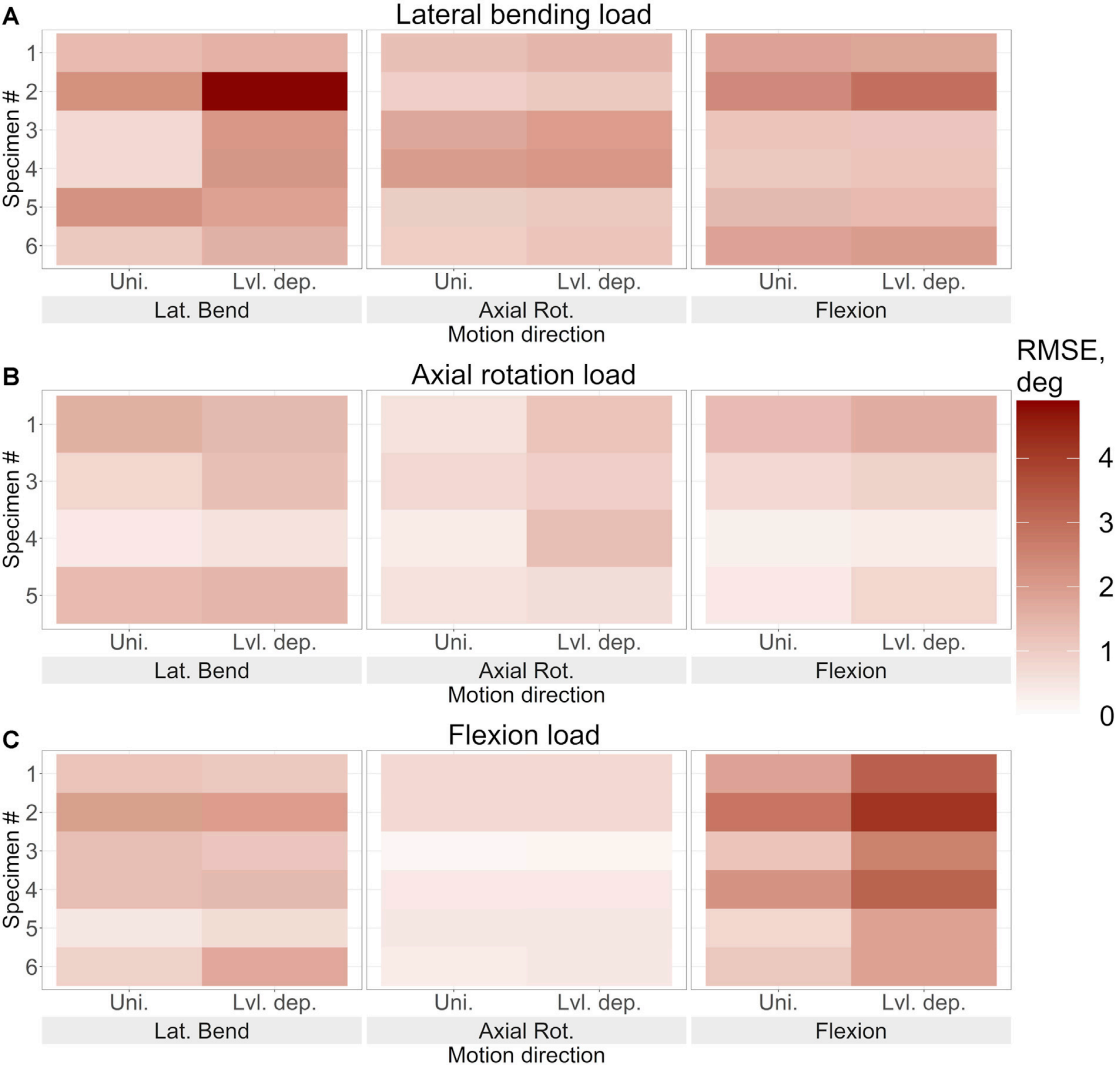
The root mean square error of the predicted motion under **(A)**. Lateral bending loading, **(B)**. Axial rotation loading, and **(C)**. Flexion loading for each specimen using the optimised stiffness. The error in lateral bending, axial rotation, and flexion is reported for uniform and level-dependent stiffness models.

The introduction of level dependency via the use of a fixed scaling factor resulted in larger prediction errors at each joint level compared to the results from models without level dependency (Figure 9). The RMSE is higher in the DoF in which the model was being loaded.

Despite the errors for both methods being substantial, significant differences were very limited. The *p-value* was less than 0.05 in axial rotation under axial rotation loading, in flexion under flexion loading, and overall under flexion loading however the *H* values were not above the *H critical* value (Table 7). The *p-value* being less than 0.05 indicates that the differences may be significant, however, as the *H* value does not surpass *H-critical* the differences may not be significant enough to reject the null hypothesis.

**TABLE 7:** Results from the Kruskal-Wallis test comparing the error in each direction and overall between the uniform and level-dependent models under each loading condition. For *p* < 0.05 the H values and the critical H values (H_c_) are also reported, for a result to be statistically significant *p* < 0.05 and H > H_c._

Loading direction	Motion direction			
	Lateral	Axial	Flexion	Overall RMSE
Lateral	NS	NS	NS	NS
Axial	NS	*p* = 0.04, H = 4.1, H_c_ = 6.0	NS	NS
Flexion	NS	NS	*p* = 0.04 H = 4.3, H_c_ = 6.0	*p* = 0.04, H = 4.3, H_c_ = 6.0

## 4 Discussion

This study aimed to investigate the inter-subject variation and the difference between spinal levels under different loading conditions. Six specimen-specific models were constructed and employed to simulate left lateral bending, left axial rotation, and flexion experiments. The IVJ stiffness was optimised with an optimisation algorithm which minimised the predicted motion error. The optimisation was performed considering (i) no level dependency, and (ii) level dependency implemented as a fixed ratio between the IVJ levels.

This study built on previous studies that have sought to determine subject-specific stiffness properties in two degrees of freedom through optimisation (Silvestros et al., 2019; Wang et al., 2021) by simultaneously optimising the stiffness in all three rotational degrees of freedom simultaneously. Rather than using data from *in vivo* experiments which require optimisations to calculate the joint kinematics, this study used *ex vivo* dataset of the lumbar spine which provides more accurate motion tracking data and removing the need for an optimisation to calculate the joint kinematics (Wang et al., 2021). Previous studies have effectively used ratios to distribute the motion across the IVJ levels (Bruno et al., 2015; Burkhart et al., 2020; Wang et al., 2021), the present study attempted to distribute the stiffness properties across the IVJ levels with a similar approach which to the best of the Authors’ knowledge has not previously been attempted.

Within the general population, a large variation of intervertebral joint stiffness is expected between individuals. Optimising stiffnesses using the tracking error reflected this, as a wide range of optimised stiffnesses were calculated (Figure 5). These stiffnesses fell within the range of experimentally reported stiffnesses (Andersson and Schultz, 1979; McGlashen et al., 1987; Garges et al., 2008; Palanca et al., 2020). This implies that the proposed optimisation approach was effective at identifying specimen-specific stiffnesses and able to capture the inter-specimen differences.

For the models without level dependency, the optimisation tended to average out the errors across the spine (observed when generic stiffness values were implemented) and the resulting stiffness was representative of an overall spinal stiffness for each specimen in each loading condition. Thus, similar kinematic errors were observed across all spine levels. Despite assuming no level dependency, substantial improvements were seen between the literature and optimised stiffnesses for each specimen (Figures 6–8). Similarly, when using level-dependent stiffnesses the optimiser was attempting to average out the errors across the levels; however, the distribution of the stiffnesses across the joint levels was defined by a fixed ratio. The level-dependent models with optimised stiffnesses were associated with markedly smaller tracking errors (Figures 6–8) compared to level-dependent models with literature stiffnesses. This supports the findings of the study by Wang *et al.* (Wang et al., 2021), that specimen-specific stiffness (level-dependent stiffnesses or uniform) is needed to accurately predict the kinematics. Furthermore, the presence of significant differences in the accuracy of the cross-validation models supports the need for specimen or subject-specific properties. Additionally, the variation of the kinematic accuracy between optimised stiffness models indicates factors other than the stiffness should also be considered, such as joint pose (Senteler et al., 2018; Byrne et al., 2020).

The optimisation of the uniform stiffnesses across spinal levels (Models_Optim_Unif_) resulted in similar errors at all spinal levels for some specimens while for other specimens there were high errors (>2°) at some levels and small errors (<0.1°), although statistically significant differences were not found. This could suggest the extent of the level dependency varies between specimens.

The extent of the motion is largely dependent on the IVJ stiffness, which has been reported to vary between spine levels (Pintar et al., 1992; Renner et al., 2007). The initial stiffnesses were based on literature data for the L3L4 IVJ level, yet the errors at the L3L4 IVJ level were comparable to the errors at the other levels when looking at the generic stiffnesses (Models_Lit_Unif_). This suggests that using a generic but level-dependent stiffness may not offer any improvement over a generic uniform stiffness in the prediction accuracy. In the present study, introducing a generic but level-dependent stiffness resulted in higher errors than when a uniform stiffness was used (Figure 9). Therefore, the introduction of level dependency via a fixed scaling factor is not suitable. However, this could also be due to the specific scaling factors used which were more suitable for some joint levels (resulting in very low prediction errors, <0.5°) and less so for other joint levels, typically the extreme joints (resulting in comparatively larger errors). The scaling factors were calculated from data presented in the meta-analysis by Zhang *et al.* (Zhang et al., 2020) (maximum rotations without a compressive preload), and the moments reported were higher than the moments used in the experiments the current study simulated. Therefore, if a ratio is used to describe the variation of the stiffness between levels, it needs to be calibrated for the loading conditions.

Considering the influence of the loading conditions, the predicted stiffnesses varied greatly for the different loading conditions. The predicted kinematic errors were markedly higher for the level-dependent models with literature stiffness under a lateral bending load, this implies the scaling ratio introduced to define the level dependency was more inappropriate for lateral bending than for axial rotation and flexion. This could indicate that the level dependency changes with the loading condition.

For all loading conditions, the stiffnesses were optimised simultaneously in all three rotational DoF for each loading condition. This is necessary as the spine exhibits coupled behaviour (Wu et al., 2014). The study by Meng *et al.* found that either coupled or uncoupled stiffnesses could represent the spinal properties however coupled and uncoupled stiffnesses should not be used interchangeably (Meng et al., 2015). In the present study, the spinal stiffnesses were modelled as uncoupled, resulting in a limited accuracy of the models in the DoF in which the load was not applied. Although the stiffnesses were optimised in these DoF their value did not change much relative to the initially set values, which were based on the literature (Table 3, Supplementary Data SB). This could be due to the motion being smaller in the unloaded directions, thus reducing the sensitivity of the cost function to these directions. Future research should address this limitation by either introducing coupling terms or by identifying a cost function that results in a better representation of the spinal stiffness in the DoF in which the load is not applied.

### 4.1 Limitations

One limitation of the study was that the analysis of the possible influence of sex was limited because of the small sample size with only a single male specimen (Table 1). However, a simple comparison of prediction errors and optimized stiffnesses did not suggest sex influenced the results, current literature is inconclusive on the relation between sex and the intervertebral disc properties (Kurutz, 2006; Mohan and Huynh, 2019; Menon et al., 2024). Furthermore, the small sample size limits the possible insights into the distribution of the IVJ stiffness properties within the population. Large datasets of human cadaveric are challenging to obtain, an alternative approach could be to use data from many separate studies (not without its own challenges in terms of data availability and consistency between studies) or to employ Monte Carlo techniques to investigate possible sex related dependencies and the distribution within the population. Additionally, the present study was limited by using a generic ratio to introduce a level dependency, this was done to reduce computational expense. The optimisation had a cardinality of three, had subject-specific level dependency been introduced the cardinality would have increased to 18 (three DoF per IVJ level), which would likely have resulted in much longer simulation times. Future research could look to use different optimisation approaches (for example, with static optimisation) to reduce computation time and allow for the higher cardinality or single functional spinal units could be investigated at different levels. Additionally, the simulations were not dynamic and did not attempt to account for the non-linearity of the joint stiffness. Given the low loading rate and the high computational expense of running fully dynamic simulations with non-linear stiffnesses, this seems to be a justifiable simplification. This study did not seek to optimise the stiffnesses in the translational DoF as the dataset contained the rotation of the vertebrae but not the translations. However, doing so would allow for a more complete characterization of the IVJ and would be a natural avenue for further work. Finally, the data used was not collected with the intention of simulating the experiments, therefore the X-rays were taken for qualitative purposes. Therefore, as the registration was based on the X-ray image, the accuracy could only be assessed qualitatively, potentially resulting in joint pose errors.

### 4.2 Conclusions

In conclusion, this study has shown that optimisation of the intervertebral joint stiffness can characterise the expected inter-specimen variability and result in more accurate motion prediction. Using a generic ratio to account for the difference of stiffnesses between spine levels results in inaccurately predicted motion, therefore specimen or subject-specific level dependency should be used to achieve more accurate predictions. Finally, the optimised stiffnesses vary widely depending on the loading direction, therefore an optimised stiffness should not only be considered specimen-specific but also load-specific.

## Data Availability

The raw data supporting the conclusion of this article will be made available by the authors, without undue reservation.
