# Supplementary material for: Variability of intervertebral joint stiffness between specimens and spine levels
Source: Front Bioeng Biotechnol. 2024 Feb 29;12:1372088. doi: 10.3389/fbioe.2024.1372088 (PMC10937554; doi:10.3389/fbioe.2024.1372088)
Supplement: Supplementary file 1 [file DataSheet2.pdf]

Supplementary Material B

Variability of intervertebral joint stiffness between specimens and spine levels

Samuele L. Gould[1,2], Giorgio Davico[1,2], Christian Liebsch[3], Hans-Joachim Wilke[3], Luca Cristofolini[1*], Marco Viceconti[1,2]

*** Correspondence:** Prof. Luca Cristofolini: luca.cristofolini@unibo.it

1 Predicted stiffness in directions which were not loaded

Plots of the optimised stiffnesses for the level-dependent models in the unloaded directions for each of the different loading conditions.

1.1 Lateral loading condition

1.1.1 Axial rotation stiffness

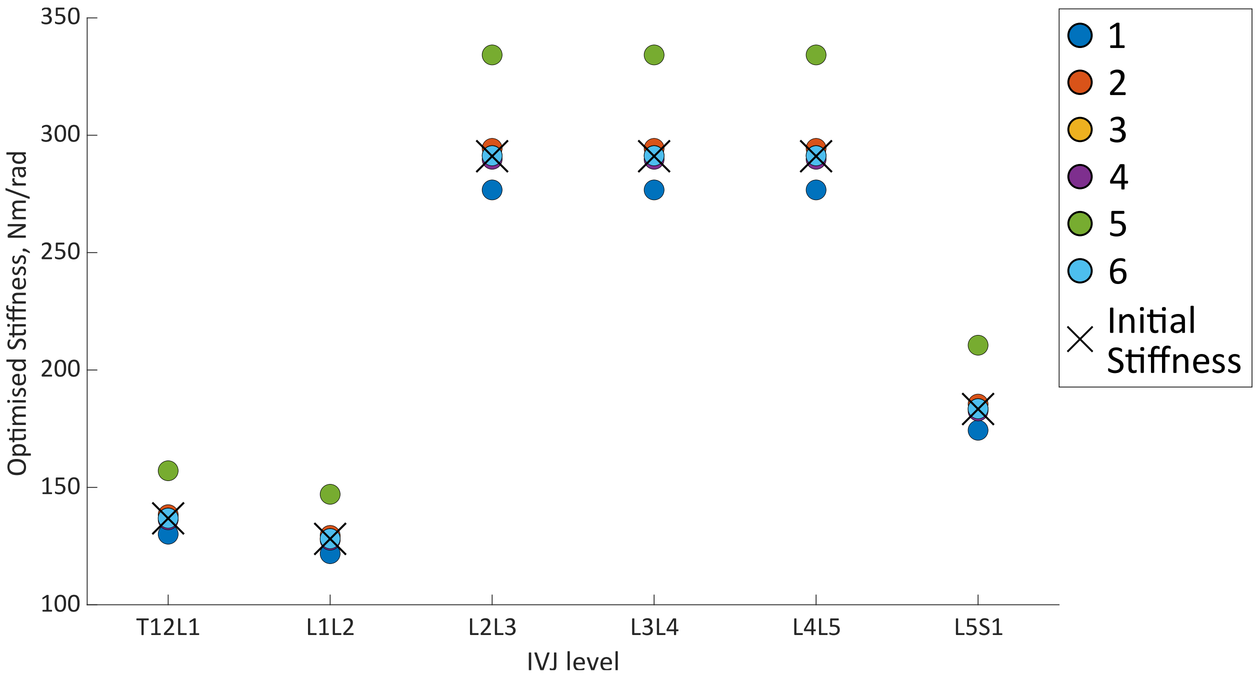

Supplementary Figure B. 1: Optimised stiffness in axial rotation for the level-dependent models under a lateral bending load.

1.1.2 Flexion stiffness

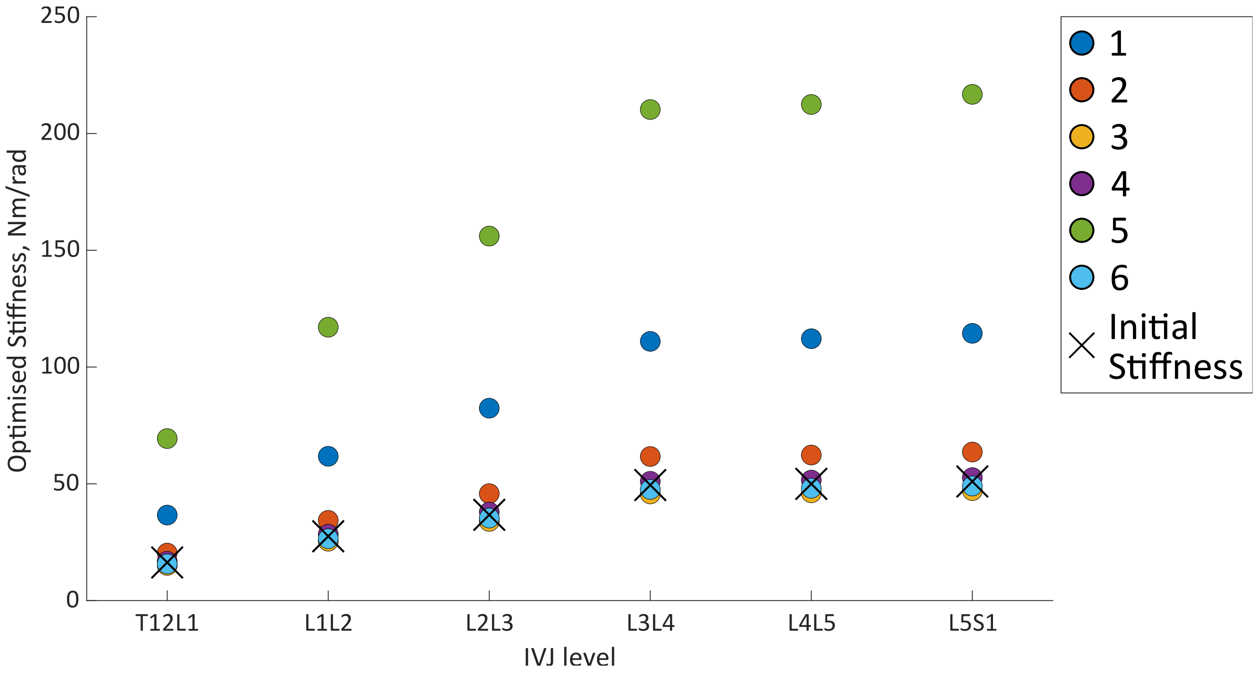

Supplementary Figure B. 2: Optimised stiffness in flexion-extension for the level-dependent models under a lateral bending load.

1.2 Axial loading condition

1.2.1 Lateral bending stiffness

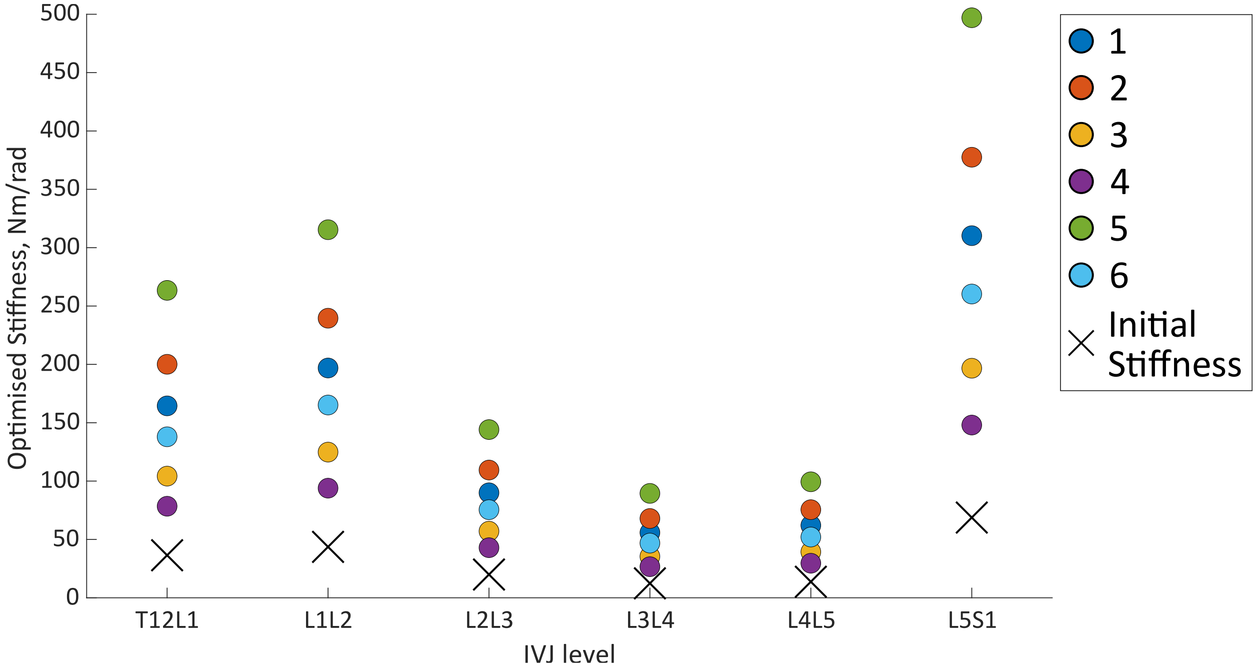

Supplementary Figure B. 3: Optimised stiffness in lateral bending for the level-dependent models under an axial rotation load.

1.2.2 Flexion stiffness

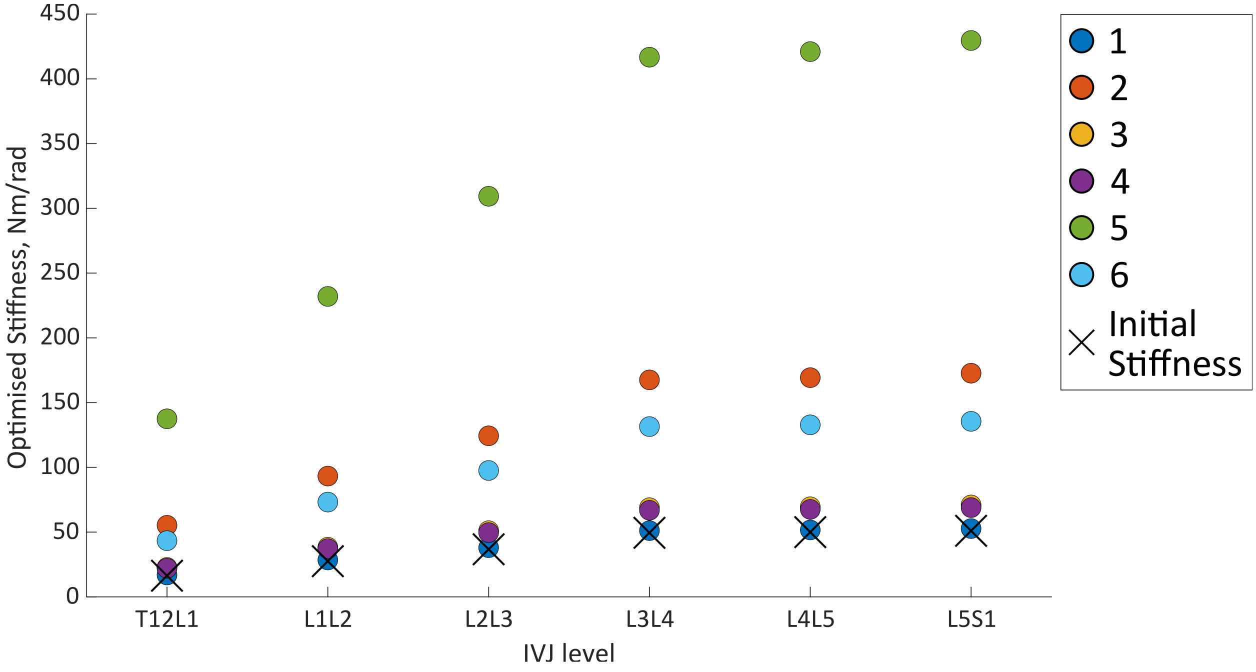

Supplementary Figure B. 4: Optimised stiffness in flexion-extension for the level-dependent models under an axial rotation load.

1.3 Flexion loading condition

1.3.1 Lateral bending stiffness

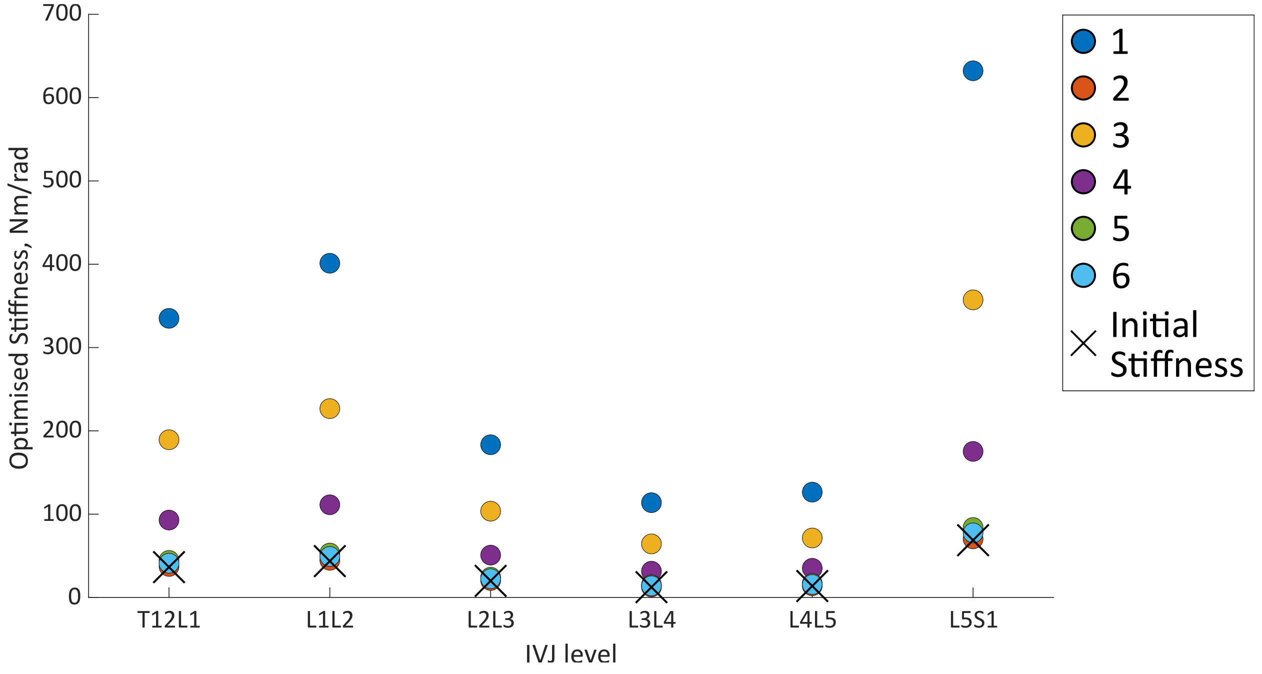

Supplementary Figure B. 5: Optimised stiffness in lateral bending for the level-dependent models under a flexion load.

1.3.2 Axial rotation stiffness

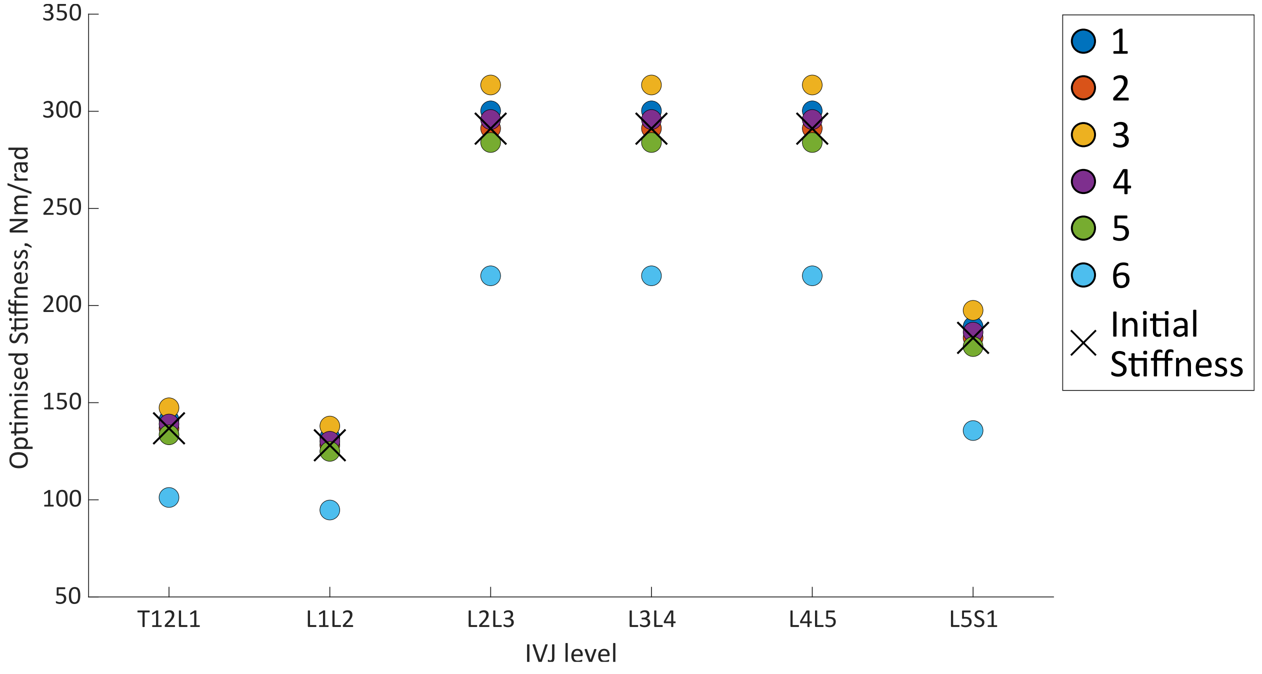

Supplementary Figure B. 6: Optimised stiffness in axial rotation for the level-dependent models under a flexion load.
