# Supplementary material for: Variability of intervertebral joint stiffness between specimens and spine levels
Source: Front Bioeng Biotechnol. 2024 Feb 29;12:1372088. doi: 10.3389/fbioe.2024.1372088 (PMC10937554; doi:10.3389/fbioe.2024.1372088)
Supplement: Supplementary file 2 [file DataSheet3.pdf]

Supplementary Material C

Variability of intervertebral joint stiffness between specimens and spine levels

Samuele L. Gould[1,2], Giorgio Davico[1,2], Christian Liebsch[3], Hans-Joachim Wilke[3], Luca Cristofolini[1*], Marco Viceconti[1,2]

*** Correspondence:** Prof. Luca Cristofolini: luca.cristofolini@unibo.it

1 Predicted errors in directions which were not loaded

Plots of the prediction errors in the DoFs which do not correspond to the loading direction. Each plot shows the error at each level for the literature stiffness model, the optimised stiffness model, and the cross-validation stiffness model.

1.1 Lateral bending loading

1.1.1 Uniform stiffnesses

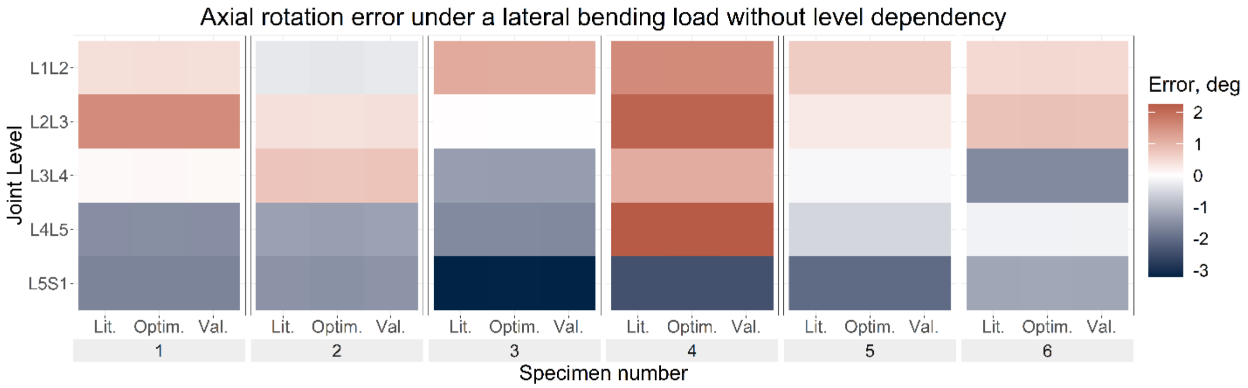

Supplementary Figure C. 1: Error of the predicted motion in axial rotation under a lateral bending load with uniform stiffnesses for the literature (Lit.), optimised (Optim.) and cross-validation (Val.) stiffnesses. Blue indicates an overprediction of the motion (ie too much bending) while red indicates an underprediction of the motion.

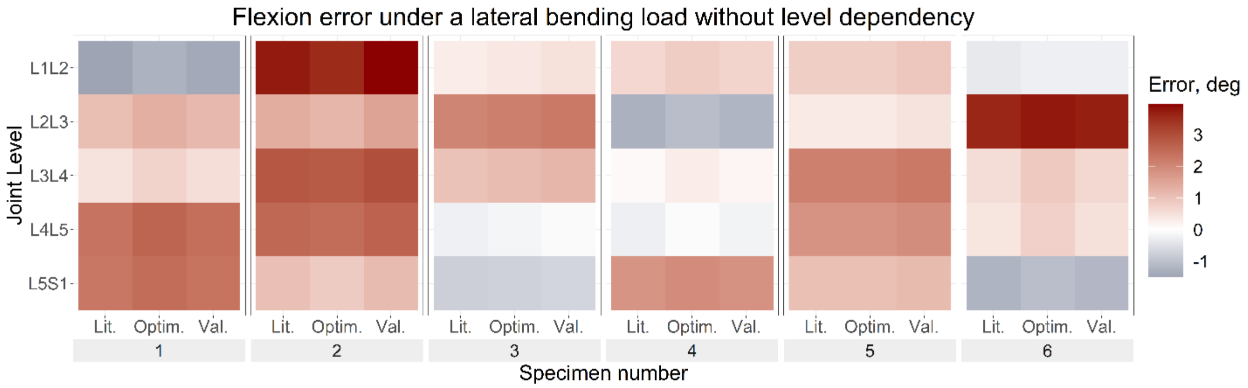

Supplementary Figure C. 2: Error of the predicted motion in flexion-extension under a lateral bending load with uniform stiffnesses for the literature (Lit.), optimised (Optim.) and cross-validation (Val.) stiffnesses. Blue indicates an overprediction of the motion (ie too much bending) while red indicates an underprediction of the motion.

1.1.2 Level-dependent stiffnesses

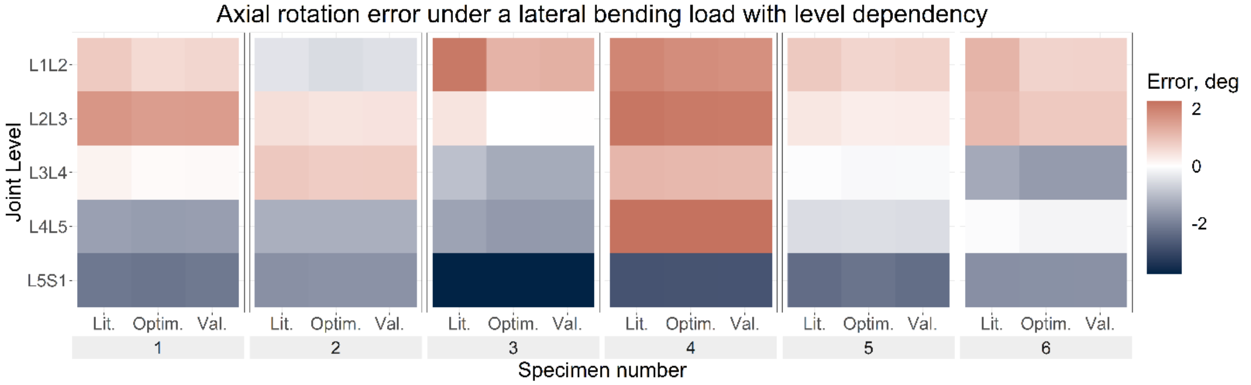

Supplementary Figure C. 3: Error of the predicted motion in axial rotation under a lateral bending load with level-dependent stiffnesses for the literature (Lit.), optimised (Optim.) and cross-validation (Val.) stiffnesses. Blue indicates an overprediction of the motion (ie too much bending) while red indicates an under-prediction of the motion.

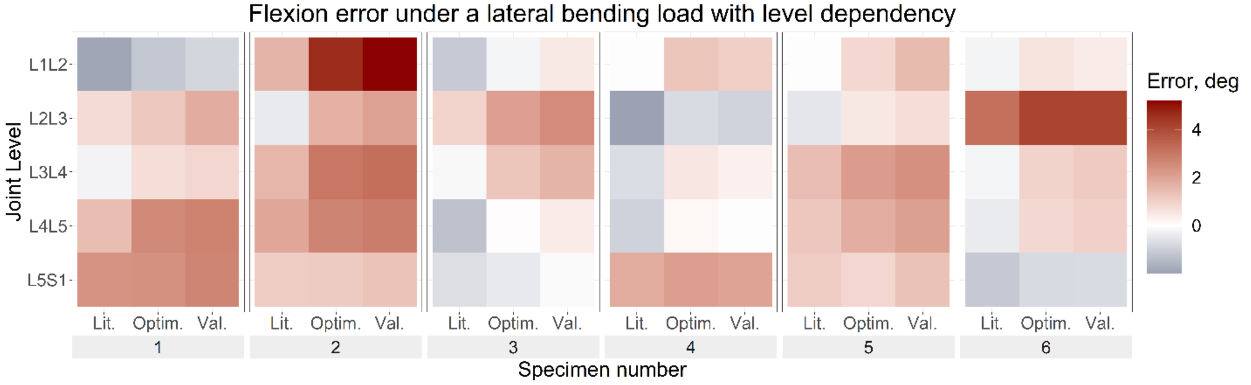

Supplementary Figure C. 4: Error of the predicted motion in flexion-extension under a lateral bending load with level-dependent stiffnesses for the literature (Lit.), optimised (Optim.) and cross-validation (Val.) stiffnesses. Blue indicates an overprediction of the motion (ie too much bending) while red indicates an underprediction of the motion.

1.2 Axial rotation loading

1.2.1 Uniform stiffnesses

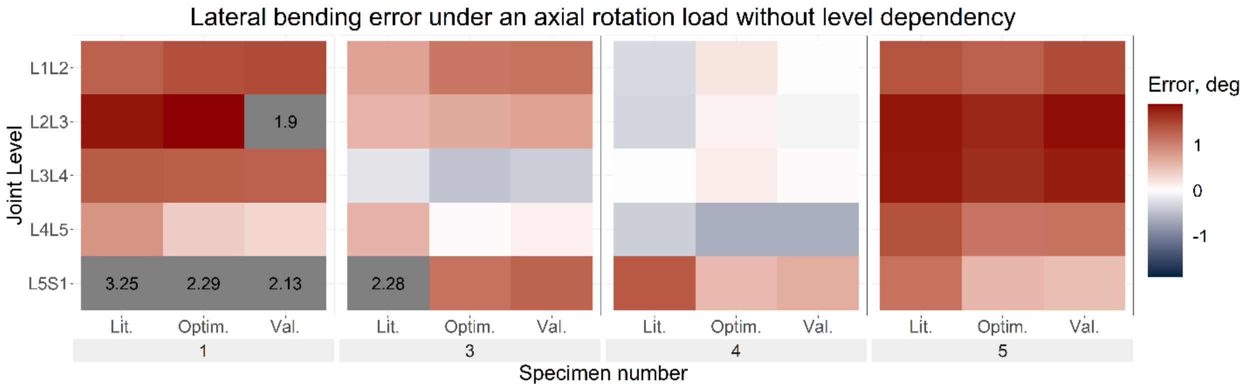

Supplementary Figure C. 5: Error of the predicted motion in lateral bending under an axial rotation load with uniform stiffnesses for the literature (Lit.), optimised (Optim.) and cross-validation (Val.) stiffnesses. Blue indicates an over-prediction of the motion (ie too much bending) while red indicates an under-prediction of the motion, and grey indicates levels where the error exceeded the range imposed on the colour scale for clarity.

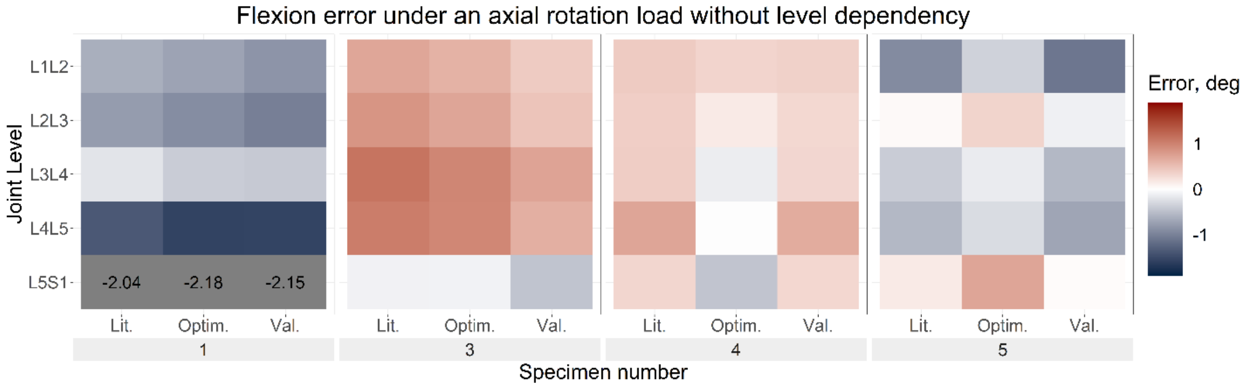

Supplementary Figure C. 6: Error of the predicted motion in flexion-extension under an axial rotation load with uniform stiffnesses for the literature (Lit.), optimised (Optim.) and cross-validation (Val.) stiffnesses. Blue indicates an overprediction of the motion (ie too much bending) while red indicates an underprediction of the motion, and grey indicates levels where the error exceeded the range imposed on the colour scale for clarity.

1.2.2 Level-dependent stiffnesses

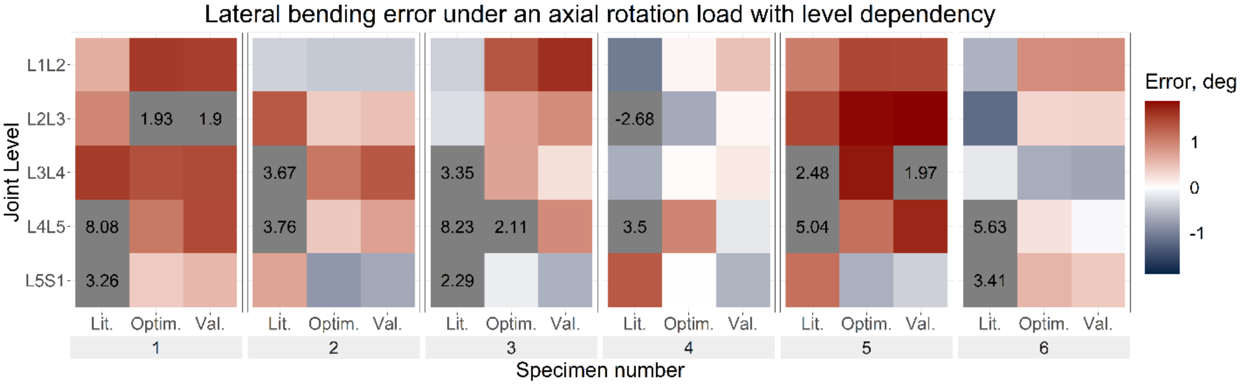

Supplementary Figure C. 7: Error of the predicted motion in lateral bending under an axial rotation load with level-dependent stiffnesses for the literature (Lit.), optimised (Optim.) and cross-validation (Val.) stiffnesses. Blue indicates an overprediction of the motion (ie too much bending) while red indicates an underprediction of the motion, and grey indicates levels where the error exceeded the range imposed on the colour scale for clarity.

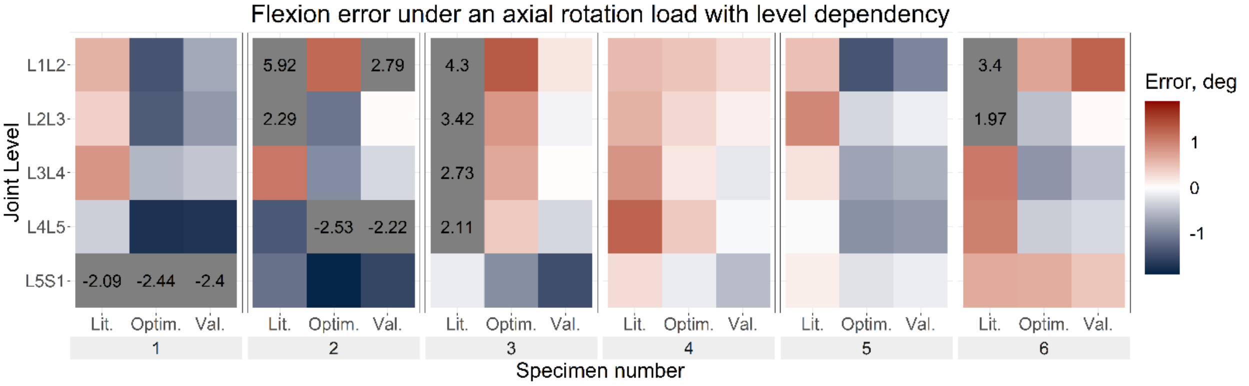

Supplementary Figure C. 8: Error of the predicted motion in flexion-extension under an axial rotation load with level-dependent stiffnesses for the literature (Lit.), optimised (Optim.) and cross-validation (Val.) stiffnesses. Blue indicates an overprediction of the motion (ie too much bending) while red indicates an underprediction of the motion, and grey indicates levels where the error exceeded the range imposed on the colour scale for clarity.

1.3 Flexion loading

1.3.1 Uniform stiffnesses

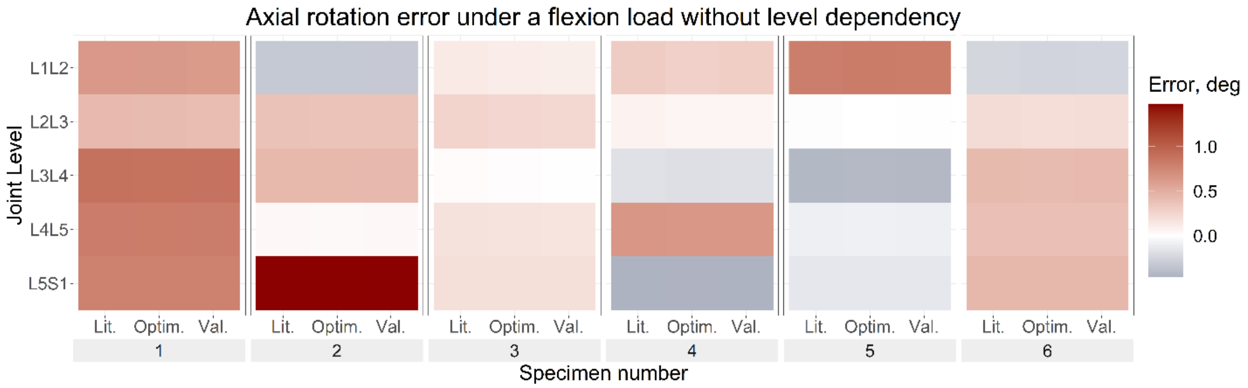

Supplementary Figure C. 9: Error of the predicted motion in axial rotation under a flexion load with uniform stiffnesses for the literature (Lit.), optimised (Optim.) and cross-validation (Val.) stiffnesses. Blue indicates an overprediction of the motion (ie too much bending) while red indicates an underprediction of the motion.

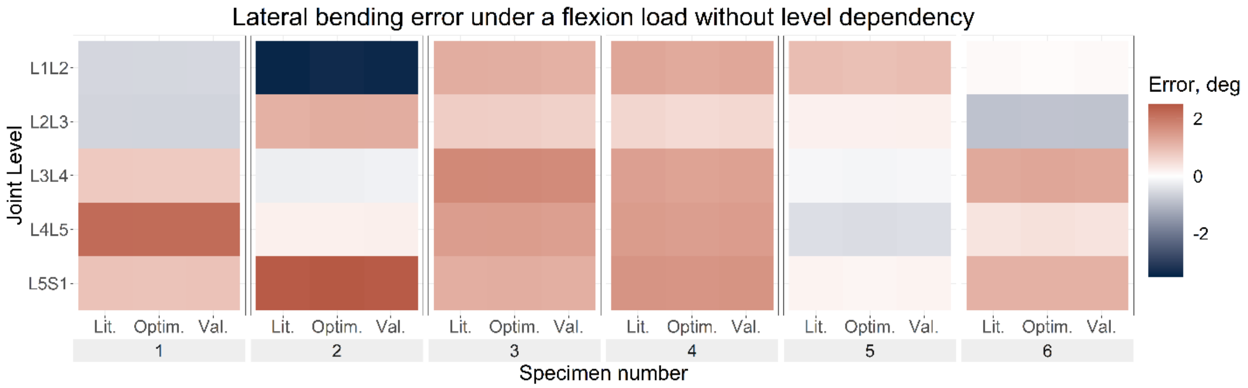

Supplementary Figure C. 10: Error of the predicted motion in lateral bending under a flexion load with uniform stiffnesses for the literature (Lit.), optimised (Optim.) and cross-validation (Val.) stiffnesses. Blue indicates an overprediction of the motion (ie too much bending) while red indicates an underprediction of the motion.

1.3.2 Level-dependent stiffnesses

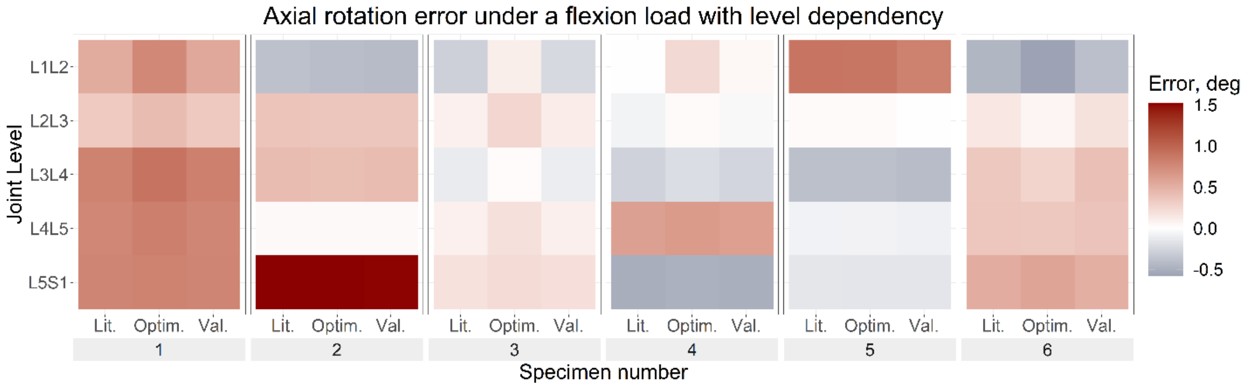

Supplementary Figure C.11: Error of the predicted motion in axial rotation under a flexion load with level-dependent stiffnesses for the literature (Lit.), optimised (Optim.) and cross-validation (Val.) stiffnesses. Blue indicates an overprediction of the motion (ie too much bending) while red indicates an underprediction of the motion.

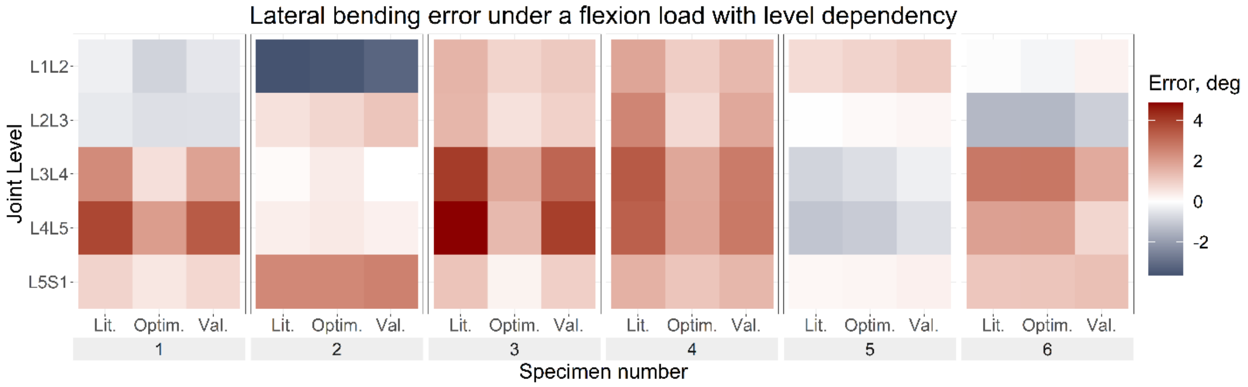

Supplementary Figure C.12: Error of the predicted motion in lateral bending under a flexion load with level-dependent stiffnesses for the literature (Lit.), optimised (Optim.) and cross-validation (Val.) stiffnesses. Blue indicates an overprediction of the motion (ie too much bending) while red indicates an underprediction of the motion.
