# Supplementary material for: Variability of intervertebral joint stiffness between specimens and spine levels
Source: Front Bioeng Biotechnol. 2024 Feb 29;12:1372088. doi: 10.3389/fbioe.2024.1372088 (PMC10937554; doi:10.3389/fbioe.2024.1372088)
Supplement: Supplementary file 3 [file DataSheet1.PDF]

Supplementary Material A

Variability of intervertebral joint stiffness between specimens and spine levels

Samuele L. Gould[1,2], Giorgio Davico[1,2], Christian Liebsch[3], Hans-Joachim Wilke[3], Luca Cristofolini[1*], Marco Viceconti[1,2]

1 Registration Process

The registration of the CT data to the X-ray can be considered in two distinct steps. First, the pre-processing of the CT data, followed by the registration of the CT data to the experimental position.

Pre-processing of CT data

Outputs:

1. A virtual palpation marker set for defining the sagittal plane of the CT data.
2. A virtual palpation marker set for defining intervertebral joints.
3. A virtual palpation marker set for defining the endplate angles.
4. Segmented specimen

Steps:

1. Perform a virtual palpation of the marker set to define the sagittal plane of the CT data. On the CT data, virtually palpate the posterior-most part of the spinous process, the anterior-most part

of the superior and inferior endplates of each vertebra, and the most inferior part of the sacrum and the process of S1, S2, S3, and apex of the coccyx (Supplementary Figure A. 1).

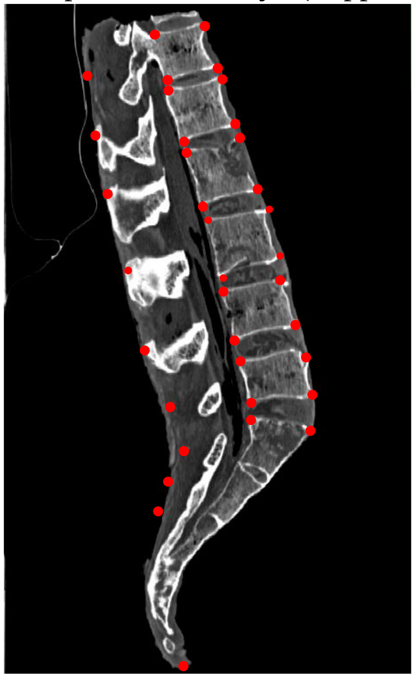

Supplementary Figure A. 1: Virtual palpation of the marker set to define the sagittal plane of the CT data.

2. Fit a plane to these points based on the least squares normal distance and calculate the necessary rotations to align the plane with the vertical.
3. Apply these rotations to the CT data (this can be done in Mimics for example with the Reslice tool)
4. Segment the vertebrae and sacrum in the CT data (Supplementary Figure A. 2).
5. Perform virtual palpation for the joint markers and the endplate angles (Supplementary Figure A. 2).

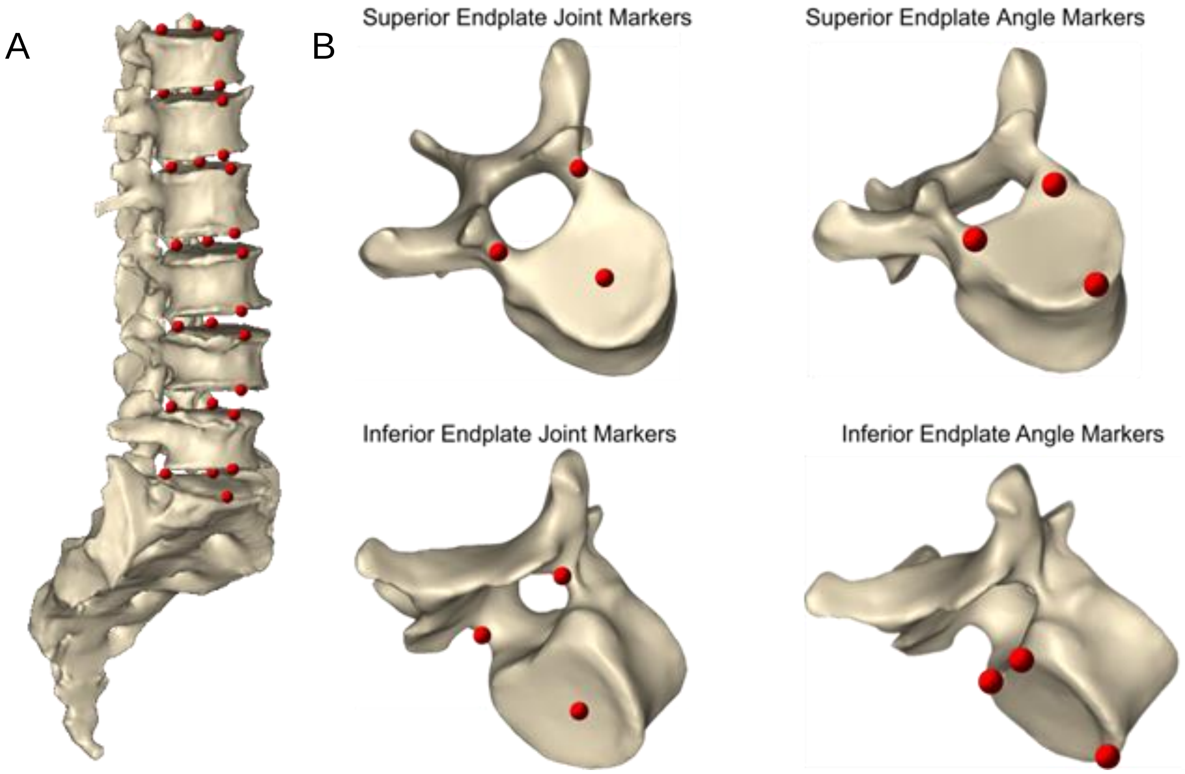

Supplementary Figure A. 2: A. Segmented specimen with markers for calculating the endplate angle. Centre – Exploded view of markers for defining the joint pose. B. Exploded view of markers for defining the endplate angle (the CT registration landmarks).

CT data registration to the X-ray

1. Perform a virtual palpation on the X-ray and calculate the endplate angles (Supplementary Figure A. 3).

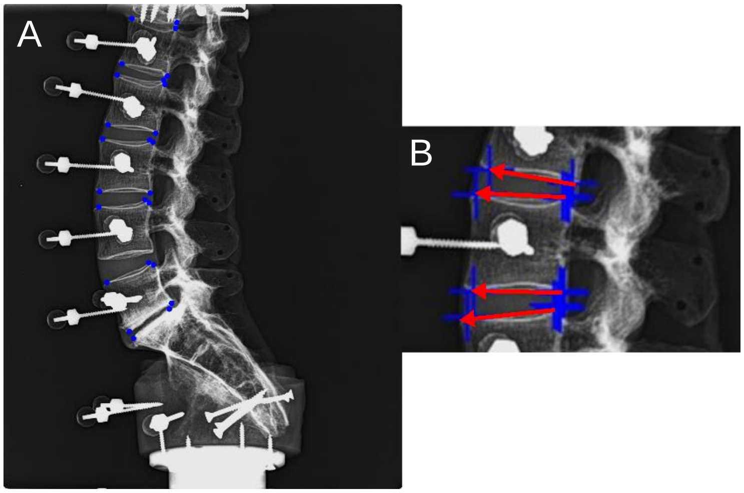

Supplementary Figure A. 3: A. Virtual palpation of the endplate on the X-ray and calculation of the endplate angles. B. determining the endplate angles.

2. Move the CT data and X-ray into a common reference system based on the sacral slope. Find the rotation to align the sacral slope of the CT to the X-ray and apply it to all the markers in the CT data (Supplementary Figure A. 4).

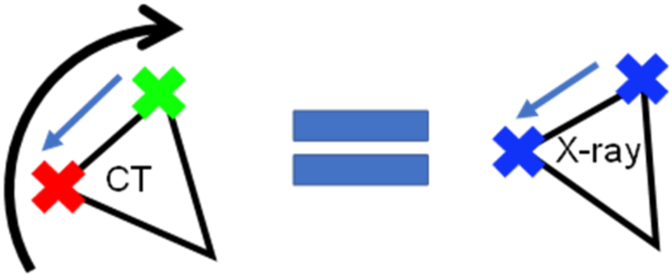

Supplementary Figure A. 4: Representation of finding the rotations to align the sacrum in the CT data with the sacrum in the X-ray.

3. Scale the X-ray to the CT data based on the average Euclidean distances of each vertebra's body height and width.
4. Calculate the endplate angles on the CT data and the rotation matrix to rotate the CT endplate angles to the X-ray endplate angles.
5. Using endplates angle markers calculate the centre of the vertebrae and the translations to move the CT vertebrae centres onto the X-ray vertebrae centres.
6. Form the transformation matrix for each vertebra from the translations and rotations and apply it to the virtual palpation markers on the CT data and the segmented geometries.
